# Prostate cancer extracellular vesicles mediate intercellular communication with bone marrow cells and promote metastasis in a cholesterol‐dependent manner

**DOI:** 10.1002/jev2.12042

**Published:** 2020-12-31

**Authors:** Stephen E. Henrich, Kaylin M. McMahon, Michael P. Plebanek, Andrea E. Calvert, Timothy J. Feliciano, Samuel Parrish, Fabio Tavora, Anthony Mega, Andre De Souza, Benedito A. Carneiro, C. Shad Thaxton

**Affiliations:** ^1^ Department of Urology Feinberg School of Medicine Northwestern University Chicago Illinois USA; ^2^ Simpson Querrey Institute for BioNanotechnology Northwestern University Chicago Illinois USA; ^3^ Robert H. Lurie Comprehensive Cancer Center Northwestern University Chicago Illinois USA; ^4^ Warren Alpert Medical School Brown University Providence Rhode Island USA; ^5^ Lifespan Cancer Institute Providence Rhode Island USA; ^6^ Department of Pathology Messejana Heart and Lung Hospital Fortaleza Brazil

**Keywords:** bone, cholesterol, exosomes, extracellular vesicles, metastasis, myeloid, pre‐metastatic niche, prostate cancer

## Abstract

Primary tumours can establish long‐range communication with distant organs to transform them into fertile soil for circulating tumour cells to implant and proliferate, a process called pre‐metastatic niche (PMN) formation. Tumour‐derived extracellular vesicles (EV) are potent mediators of PMN formation due to their diverse complement of pro‐malignant molecular cargo and their propensity to target specific cell types (Costa‐Silva et al., 2015; Hoshino et al., 2015; Peinado et al., 2012; Peinado et al., 2017). While significant progress has been made to understand the mechanisms by which pro‐metastatic EVs create tumour‐favouring microenvironments at pre‐metastatic organ sites, comparatively little attention has been paid to the factors intrinsic to recipient cells that may modify the extent to which pro‐metastatic EV signalling is received and transduced. Here, we investigated the role of recipient cell cholesterol homeostasis in prostate cancer (PCa) EV‐mediated signalling and metastasis. Using a bone metastatic model of enzalutamide‐resistant PCa, we first characterized an axis of EV‐mediated communication between PCa cells and bone marrow that is marked by in vitro and in vivo PCa EV uptake by bone marrow myeloid cells, activation of NF‐κB signalling, enhanced osteoclast differentiation, and reduced myeloid thrombospondin‐1 expression. We then employed a targeted, biomimetic approach to reduce myeloid cell cholesterol in vitro and in vivo prior to conditioning with PCa EVs. Reducing myeloid cell cholesterol prevented the uptake of PCa EVs by recipient myeloid cells, abolished NF‐κB activity and osteoclast differentiation, stabilized thrombospondin‐1 expression, and reduced metastatic burden by 77%. These results demonstrate that cholesterol homeostasis in bone marrow myeloid cells regulates pro‐metastatic EV signalling and metastasis by acting as a gatekeeper for EV signal transduction.

## 1 INTRODUCTION

Extracellular vesicles (EV) are particles released by cells that are delimited by a lipid bilayer and incapable of replication (Thery et al., 2018). Sub‐types of EVs include endosome‐originating exosomes, plasma membrane‐derived ectosomes or microvesicles, large oncosomes, apoptotic bodies, and others. Small EVs (sEV), on the order of 30–200 nm in diameter, are often released in abundance by neoplastic cells and have been shown to promote metastasis by communicating with cells at distant, pre‐metastatic organ sites to create tumour‐favouring microenvironments, a process called pre‐metastatic niche (PMN) formation (Lobb, Lima, & Moller, 2017; Peinado et al., 2017). This phenomenon has been found to occur in melanoma (Peinado et al., 2012), pancreatic cancer (Costa‐Silva et al., 2015), prostate cancer (Dai et al., 2019), and other tumour types. Comparatively little attention has been paid, however, to the factors intrinsic to recipient cells that may influence the ability of tumour‐derived EVs to efficiently mediate PMN formation. Here, we investigate the role of recipient cell cholesterol homeostasis in regulating cancer EV‐mediated signalling and metastasis in the setting of prostate cancer (PCa).

PMN formation requires long‐range intercellular communication to be persistently mediated by soluble or membrane‐bound factors that originate from the primary tumour (Karnezis et al., 2012; Liu & Cao, 2016; Peinado et al., 2017). After traversal of vascular and interstitial spaces, these factors eventually engage with the cholesterol‐rich plasma membrane of recipient cells at pre‐metastatic organ sites. The synaptic events of intercellular communication, such as ligand‐receptor interactions or endocytosis, may then lead to successful transduction of a pro‐metastatic signal, for instance by perturbing intracellular signalling and/or gene expression. These events are tightly controlled by the organization of the recipient cell's plasma membrane (Bethani, Skanland, Dikic, & Acker‐Palmer, 2010; Fessler & Parks, 2011) which, in turn, is regulated by cholesterol. Cholesterol comprises approximately 40 mol% of mammalian plasma membrane lipids (Steck & Lange, 2018), and alters membrane organization by influencing protein scaffolding (Epand, Sayer, & Epand, 2005; Paila & Chattopadhyay, 2010; Sheng et al., 2012), lipid raft stability (Lajoie & Nabi, 2010; Simons & Ehehalt, 2002), and the cohesion and orientation of membrane phospholipids (Martinez‐Seara, Rog, Karttunen, Vattulainen, & Reigada, 2010). In addition, cholesterol is required for the structure of clathrin and caveolin‐dependent membrane invaginations, and lipid raft‐dependent signalling and endocytosis (Hoop, Sivanandam, Kodali, Srnec, & van der Wel, 2012; Lajoie & Nabi, 2010). Hence, cholesterol is well‐poised to globally regulate the efficiency of intercellular communication in PMN formation and other settings. Moreover, our group and others have shown that perturbation of cell membrane cholesterol can inhibit uptake of EVs by recipient cells (Plebanek et al., 2015; Svensson et al., 2013). We therefore hypothesized that cellular cholesterol burden at pre‐metastatic sites may critically regulate the efficiency of cancer EV‐mediated intercellular communication with target cells at these sites.

The bone marrow compartment is a dynamic repository for triglycerides, fatty acids, and cholesterol, and is particularly sensitive to serum lipid levels (Rosen, Ackert‐Bicknell, Rodriguez, & Pino, 2009; Wang, Leng, & Gong, 2018). Hence, it represents an ideal tissue for probing lipid‐dependent intercellular communication. Moreover, bone marrow‐derived cells (BMDC) are frequently the targets of pro‐metastatic signals originating from the primary tumour, making the bone marrow compartment a hotbed for pro‐metastatic signalling and metastasis (Chow et al., 2014; Costa‐Silva et al., 2015; Liu & Cao, 2016; Peinado et al., 2012; Yu et al., 2007). Tumour‐derived EVs frequently target BMDCs and have proven to be versatile and potent mediators of PMN formation via horizontal transfer of pro‐metastatic biochemical signals (Costa‐Silva et al., 2015; Dai et al., 2019; Hoshino et al., 2015; Maas, Breakefield, & Weaver, 2017; Peinado et al., 2012; Peinado et al., 2017). Therefore, we investigated tumour‐derived EVs as mediators of pro‐metastatic signalling and identified bone marrow‐resident myeloid cells as a potential target cell population. Finally, we focused on a uniformly lethal metastatic process, namely the development of bone metastases in PCa. Approximately 90% of men who succumb to PCa are found to have bone metastasis at autopsy (Bubendorf et al., 2000). Moreover, PCa bone metastases are heavily laden with cholesterol in comparison to healthy bone and metastases of other origins (Thysell et al., 2010), making PCa bone metastasis particularly attractive for a case study in cholesterol‐dependent PMN formation.

## 2 MATERIALS AND METHODS

### 2.1 Cell cultures

All cells were maintained at 37°C, 5% CO_2_, and were handled under sterile conditions in an Esco Class II Type A2 biosafety cabinet. EnzR CWR‐R1 cells and EnzR LNCaP cells were gifts from Dr. Donald Vander Griend (University of Illinois at Chicago) and were cultured in Roswell Park Memorial Institute (RPMI) 1640 medium containing 10% FBS, 1% penicillin/streptomycin and 20 µM enzalutamide. PC3, DU145, LNCaP (non‐resistant), CWR‐R1 (non‐resistant), PNT2 and THP1‐Dual cells (ATCC, Manassis, VA) were cultured in RPMI containing 10% fetal bovine serum (FBS), and 1% penicillin/streptomycin. RAW264.1 (ATCC) cells were cultured in Dulbecco's Modified Eagle Medium (DMEM) containing 10% FBS and 1% penicillin/streptomycin. Cells were passaged upon reaching approximately 70–80% confluence.

### 2.2 Experimental animals

Mice were housed and maintained in the Northwestern University Center for Comparative Medicine, according to NIH guidelines and in concordance with protocols approved by the Northwestern University Institutional Animal Care and Use Committee (IACUC). Male C57BL/6 mice (3–6 weeks) were obtained from the Jackson Laboratory and male C.B.‐17 SCID mice (3–4 weeks) were obtained from Taconic Biosciences.

### 2.3 Bone marrow isolation

Total bone marrow was harvested from healthy male C57BL/6 mice by dissecting hind limbs immediately after euthanasia, soft tissue was removed, and bone marrow cavities of femurs and tibias were flushed with phosphate buffered saline (pH = 7.4) (PBS). Flushed cells and tissue were then passed through 70 µm filters to remove non‐cellular tissue components and debris. Cells were then centrifuged at 300 x *g* and resuspended in Red Blood Cell Lysis Buffer (Invitrogen) for 5 min to lyse RBCs followed by centrifugation at 300 x *g*.

### 2.4 Primary bone marrow macrophage culture

Bone marrow cells were harvested as described above, cells were resuspended in DMEM containing 10% FBS, 1% penicillin/streptomycin, and 20 ng/ml M‐CSF (BioLegend). Cells were then seeded in 6 cm dishes at 1 million cells/dish and macrophage differentiation proceeded for 7 days, with 5 ml of fresh, M‐CSF containing medium added at day 4.

### 2.5 EV isolation

Cells were cultured and grown into 15 cm dishes. When cells reached approximately 70–80% confluence, cells were washed with PBS and transferred to fresh media containing EV‐free serum for 48 h. Media was then collected and subjected to centrifugation at 300 x *g* for 5 min to pellet cells, then 2000 x *g* for 15 min to remove cellular debris. The supernatant was then transferred to ultracentrifuge tubes and ultracentrifuged at 10,000 x *g* for 30 min to pellet and remove large vesicles. The supernatant was then transferred to new ultracentrifuge tubes and ultracentrifuged twice at 100,000 x *g* for 90 min to pellet EVs; the EV pellet was washed with PBS between the two centrifuge steps to remove co‐pelleted non‐EV components, and then resuspended in PBS. After the final round of centrifugation, EVs were resuspended in PBS and then either stored at 4°C for short‐term use (less than 3 days) or stored at −80°C for later use. Protein content of EVs was determined by BCA and treatment dosing was determined by EV protein concentration.

### 2.6 HDL NP synthesis

High‐density lipoprotein‐like nanoparticles (HDL NPs) were synthesized according to published protocols. Briefly, particle synthesis was initiated by adding purified apolipoproteinA‐1 (apoA‐1) (MyBioSource) at fivefold molar excess to a solution of 5 nm diameter citrate‐stabilized, colloidal gold nanoparticles (Au NPs) (80–100 nM; Ted Pella, Inc). The suspension was vortexed briefly, and placed on shaker at RT for 1 h. Next, two species of phospholipid – 1,2‐dipalmitoyl‐*sn*‐glycero‐3‐phosphoethanolamine‐*N*‐[3‐(2‐pyridyldithio)propionate] (PDP PE) and 1,2‐dipalmitoyl‐*sn*‐glycero‐3‐phosphocholine (DPPC) (Avanti Polar Lipids)— were added to the suspension at 250‐fold molar excess to Au NPs in a mixture of ethanol and water (1:4) and incubated for 4 h at RT with gentle mixing on a flat‐bottom shaker. HDL NPs were then purified and concentrated using tangential flow filtration. HDL NP concentration was determined using UV‐Vis spectroscopy (ε_Au NP_ = 9.696 × 10^6^ M^–1^ cm^–1^, λ_max_ = 520 nm) and size was confirmed using dynamic light scattering.

### 2.7 Transmission electron microscopy and dynamic light scattering

EV samples were fixed by adding an equal volume of 4% paraformaldehyde (PFA) to the EV suspension and incubating at room temperature for 10 min. 5 µl of EV suspension were deposited on 300‐mesh carbon‐coated copper grids to adsorb for 20 min. Grids were then floated on 100 µl drops of PBS on parafilm for 2 min. Grids were then transferred to 50 µl drops of 1% glutaraldehyde for 5 min. Next, grids were washed on 100 µl drops of PBS for a total of 8 washes and 2 min per wash. Grids were then transferred to 50 µl drops of uranyl oxalate, pH = 7, for 5 min, and finally to 50 µl drops of methyl cellulose‐uranyl acetate for 10 min on ice. Excess fluid was then blotted off on filter paper, and grids were dried at room temperature for 20 min prior to imaging or storage. Imaging was performed using a FEI Tecnai Spirit transmission electron microscope (TEM) operating at 80 kV. Hydrodynamic diameter of EVs was determined by dynamic light scattering (DLS), which was performed using a Zetasizer Nano ZS (Malvern). EV samples were diluted to concentrations of 1–10 µg EV protein per ml in PBS. Hydrodynamic diameters reported are the average of three separate measurements of a single sample, with each measurement being the cumulative result of ten runs.

### 2.8 EV fluorescent labelling

EVs were isolated from cultured cells in an identical manner as described above, with the exception that prior to the last round of ultracentrifugation at 100,000 x *g*, the lipophilic fluorescent dye 1,1′‐Dioctadecyl‐3,3,3′,3′‐Tetramethylindocarbocyanine Perchlorate (DiI) was added to the EV sample at a concentration of 2.5 µM in PBS. After the final round of centrifugation, supernatant containing excess DiI was discarded prior to resuspension of the EV pellet.

### 2.9 Flow cytometry

DiI‐labelled EVs or vehicle control (PBS) were injected via tail vein into male C57BL/6 mice (10 µg EV protein) 24 h prior to euthanasia. For in vivo uptake inhibition experiments, mice were subjected to HDL NP injection via tail vein (100 µM, 100 µl) 24 h prior to injection of DiI‐labelled EVs. Bone marrow cells were then isolated as described above, washed in PBS, and resuspended in FACS buffer [PBS containing 1% bovine serum albumin (BSA), 0.1% sodium azide]. Samples were stained in LIVE/DEAD Aqua Dead Cell stain (ThermoFisher) for 20 min at RT, washed in FACS buffer, and then blocked in F_c_ block (BD Pharmingen) for 20 min at RT. Cells were then incubated in 100 µl of fluorophore‐conjugated antibody cocktail (APC anti‐mouse/human CD11b, PE‐Cy7 anti‐mouse Ly6C, Brilliant Violet anti‐mouse Ly6G; BioLegend) (1:100 dilutions for all antibodies) for 1 h at 4°C protected from light, and then washed in FACS buffer. Samples were then analyzed using a BD LSR Fortessa Analyzer and data was processed using FlowJo software. A sample of the gating scheme is shown in Figure S1c. Experiments were performed using *n* = 3 mice per group.

### 2.10 In vitro EV uptake studies

Cells were plated at 20,000 cells per well in 24‐well plates on top of glass coverslips, in 0.5 ml of culture medium. The following day, cells were washed three times in PBS prior to treatment with DiI‐labelled EVs with or without HDL NP pre‐treatment (2 h) in serum‐free media. For all in vitro EV uptake experiments excepting time‐dependence studies (Figure 2a and c, Figure S1a, Figure S4), EV uptake was allowed to proceed for 30 min. For time‐dependence studies, EV uptake proceeded for 30 min, 2 h, or 12 h. Cells were then washed three times in PBS and fixed in 4% paraformaldehyde for 15 min at RT. Subsequently, cells were washed three times in PBS, and incubated with AlexaFluor488‐Phalloidin (1:1000) (Abcam) and DAPI (300 nM) for 1 h at RT. Cells were then washed three times in PBS and mounted on microscopy slides in Fluoromount G mounting medium (Southern Biotech). Three distinct samples from each group were imaged using a Nikon A1R Spectral confocal microscope.

### 2.11 Osteoclast differentiation studies

For in vitro osteoclast differentiation assays, RAW264.7 cells were seeded at a density of 25,000 cells per well in 24‐well tissue culture plates. After 48 h, cells were incubated with vehicle control (PBS), EnzR EVs (5 µg/ml EV protein) with or without RANKL (20 µg/ml), or RANKL alone for 7 days, with fresh media containing EVs and/or RANKL added at day 4. For in vitro HDL NP inhibition studies, HDL NPs (100 nM) were added to the media 2 h prior to addition of EnzR EVs, and osteoclast differentiation was allowed to proceed for 7 days, with fresh media containing HDL NPs and EnzR EVs added at day 4. After 7 days, cells were then fixed in 4% PFA and stained with TRAP using a commercially available kit (Sigma) according to the manufacturer's protocol. Experiments were performed using three distinct biological replicates per group.

For in vivo osteoclast differentiation studies, C57BL/6 mice were subjected to three tail vein injections of EnzR EVs (10 µg EV protein per injection) at 48 h intervals. Mice were euthanized 24 h after the third injection, hind limbs were dissected, soft tissue was removed, and bones were fixed in 10% neutral buffered formalin (NBF) for 3–5 days. Hind limb bones were then rinsed twice in PBS, placed in 70% ethanol for 24 h, and decalcified in 20% EDTA for 10 days prior to paraffin embedding, sectioning, and mounting on microscopy slides. TRAP staining was then performed to visualize osteoclasts using a commercially available kit (Sigma) according to the manufacturer's protocol. Experiments were performed using *n* = 3 mice per group.

### 2.12 NF‐κB reporter assay

THP1‐Dual cells were maintained in culture medium containing 10 µg/ml blasticidin and 100 µg/ml Zeocin to maintain selection pressure and passaged every three days. Cells were seeded in 96‐well plates at 100,000 cells/well in 180 µl of cell suspension. Then 20 µl of EnzR EVs, vehicle control (PBS), or positive control (LPS) were added to each well to achieve the desired final concentration. Plates were incubated at 37°C for 24 h. QUANTI‐Blue solution was then prepared according the manufacturer's instructions, 180 µl of QUANTI‐Blue solution were added to each well of a new 96‐well plate, and 20 µl of THP1‐Dual supernatant were added to each well. Plates were incubated at 37°C for 2 h and SEAP levels were determined by measuring absorbance using a Synergy Plate Reader. For HDL NP inhibition experiments, the experimental conditions were identical with the exception that HDL NPs (100 nM) were added to THP1 cells 2 h prior to addition of EnzR EVs. NF‐κB activity was measured from four distinct biological replicates per group.

### 2.13 Radiolabeled cholesterol efflux assay

THP1 monocytes were seeded into 24‐well plates at a density of 50,000 cells/well and differentiated into macrophages by incubating with 100 ng/ml phorbol 12‐myristate 13‐acetate (PMA) for 48 h at 37°C. Cells were then washed three times with PBS and fresh, non‐PMA containing media was added to allow cells to recover for 24 h prior to loading with tritium‐labelled cholesterol ([^3^H]‐chol). An ethanol stock solution of [^3^H]‐chol was evaporated to generate a thin film, re‐dissolved in 1 ml ethanol and incubated at 37°C for 60 min. The solution was then evaporated again, re‐dissolved in 50 µl ethanol, and incubated at 37°C for 30 min. FBS was then added to the solution of [^3^H]‐chol at a quantity calculated to enable labelling of cells the following day with 1 µCi [^3^H]‐chol per well in 5% FBS containing culture medium. The resulting mixture was incubated at 4°C overnight. On the following day, serum‐free RPMI with 1% penicillin/streptomycin was added to the mixture of [^3^H]‐chol and FBS to yield a labelling medium comprising 2 µCi/ml [^3^H]‐chol, 5% FBS. Cells were washed three times with PBS and 500 µl of labelling medium were added to the cells. After 24 h, cells were washed three times with PBS to remove excess [^3^H]‐chol, and fresh culture medium with HDL NPs or vehicle (PBS) control was added. Cholesterol efflux was allowed to proceed for 4 h when cells were washed three times with PBS, lipids were extracted with isopropanol, and [^3^H]‐chol was quantified using liquid scintillation counting. [^3^H]‐chol was quantified from four distinct biological replicates per group.

### 2.14 In vivo bone marrow cellular cholesterol quantification studies

Male C57BL/6 were injected with HDL NPs (100 µl, 1 µM) or vehicle alone (PBS) via tail vein 2 h prior to euthanasia when hind limbs were dissected and bone marrow cells were isolated as described above. Each bone marrow sample was then divided into two groups: one for quantification of cellular cholesterol in total bone marrow cells and the other for quantification of cellular cholesterol in bone marrow monocytes. For the latter group, a commercially available mouse bone marrow isolation kit (EasySep) was used to isolate monocytes from total bone marrow. Amplex Red Cholesterol Assay (ThermoFisher) was then used to quantify cellular cholesterol in both groups according to the manufacturer's instructions. Protein content of the samples was determined by BCA, and cholesterol quantification was normalized to the protein content. Experiments were performed using *n* = 5 mice per group.

### 2.15 Immunohistochemistry

Male C57BL/6 mice were subjected to three tail vein injections of EnzR EVs (10 µg EV protein per injection) or vehicle (PBS) control at 48 h intervals. Mice were euthanized 24 h after the third injection, hind limbs were dissected, soft tissue was removed, and bones were fixed in 10% neutral buffered formalin (NBF) for 3–5 days. Hind limb bones were then rinsed twice in PBS, placed in 70% ethanol for 24 h, and decalcified in 20% EDTA for 10 days prior to paraffin embedding, sectioning, and mounting on microscopy slides. Tissues were deparaffinized and rehydrated by incubating twice in xylene for 5 min, and then undergoing successive 10‐minute incubations in 100% ethanol, 95% ethanol, 70% ethanol, distilled water and finally PBS. Tissues were then washed and permeabilized in TBS‐Triton (0.025% Triton X‐100) twice for five minutes and blocked in TBS with 1% BSA, 10% donkey serum. Primary antibodies (anti‐mouse TSP1, Abcam, 1:50 dilution; anti‐mouse/human VCAN, Sigma, 1:100 dilution; anti‐mouse Type I Collagen, Sigma, 1:100 dilution) were then added using TBS, 1% BSA as a diluent and allowed to incubate for 24 h at 4°C. Tissues were then washed twice in TBS‐Triton for 5 min each, prior to adding fluorescently tagged secondary antibodies at 1:250 dilutions in TBS, 1% BSA. Tissues were then washed with TBS for 5 min, then DAPI (300 nM) in TBS, and once more in TBS for five minutes prior to mounting and cover slipping on microscopy slides in Vectashield mounting media. Slides were imaged using a Nikon A1R spectral confocal microscope. Experiments were performed using *n* = 3 mice per group.

### 2.16 Surgical castration

Male C.B.‐17 SCID mice aged 3–4 weeks were surgically castrated two weeks prior to injection of tumour cells. Mice were anesthetized under inhaled isoflurane. The incision area was swabbed with alcohol and betadine prior to surgery. A single 2–4 mm incision was made in the inferior scrotum. One testicle was removed from the scrotum, a knot was tied around the base of the testicle, and the testicle was excised with surgical scissors. The process was repeated for the other testicle. The incision site was then closed with absorbable sutures. Mice were given intraperitoneal injections of meloxicam (5 mg/kg) once per day for two days following the operation.

### 2.17 Metastasis experiments

Male C.B.‐17 SCID mice (Taconic Bioscience) were castrated two weeks prior to intracardiac injection of EnzR CWR‐R1 cells. Five days prior to intracardiac injection, mice were subjected to three rounds of tail vein injections with EnzR EVs (10 µg EV protein per injection, 100 µl) or vehicle control (PBS) at 48 h intervals. One group of mice was also subjected to three rounds of HDL NP injections (100 µM, 100 µl) via tail vein on alternating days from EnzR EV injections, beginning 24 h prior to the first EnzR EV injection. On the day of intracardiac injection, mice were anesthetized under inhaled isoflurane, the injection site was swabbed with alcohol and betadine, the syringe needle was then advanced through the skin approximately 1 cm to the left of the inferior sternum and 5 mm inferior to the sternum. The needle was then advanced through the diaphragm and into the left ventricle, and luciferase‐expressing EnzR CWR‐R1 cells (250,000 cells) were injected into the left ventricle over the course of 15–20 s. Mice were monitored for signs of pain following the procedure and meloxicam was administered as needed. Tumour burden was assessed at 2 weeks post‐intracardiac injection via bioluminescence imaging. Mice were injected intraperitoneally with luciferin (150 mg/kg) 5 min prior to bioluminescence imaging under anaesthesia. Experiments were performed using *n* = 8 mice per group for experimental groups (EnzR EVs; EnzR EVs + HDL NP) and *n* = 5 mice were used for the PBS control group.

### 2.18 Adhesion assay

Recombinant type I collagen (Sigma), TSP1 (R&D Systems), and VCAN (Ray Biotech) were diluted to 100 µg/ml in PBS. All ECM substrates were then prepared at a final total protein concentration of 5 µg/ml using the appropriate combination of each of the three components. The remainder of the substrate that did not call for TSP1 or VCAN was filled with type I collagen. For instance, a condition that called for 5% TSP1, 45% VCAN would be prepared by adding 5 equivalents of TSP1, 45 equivalents of VCAN, and 50 equivalents of type I collagen. Substrates were then sonicated to achieve greater alignment of ECM fibrils. 100 µl of each substrate were then added in triplicate to wells in 96‐well plates and incubated for 2 h at room temperature. Excess substrates were then removed by inverting the 96‐well plate over a plastic reservoir and tapping gently. Wells were washed twice with PBS and blocked with DMEM containing 10% FBS for 30 min at 37°C. Wells were washed once with PBS and 50,000 EnzR CWR‐R1 cells were added to each well in full serum‐containing culture medium and incubated at 37°C in an IncuCyte S3 system to enable live cell imaging of the adhesion process. Cells were imaged at 15 min intervals for the first 2 h and then every hour for the next 6 h. IncuCyte Analysis software was then used to analyse the results. Statistical testing was performed using three distinct biological replicates. Each data point from a single biological replicate represents the mean confluence across five technical replicates (distinct images from five different parts of the same tissue culture well).

### 2.19 RNA isolation for sequencing

For CD11b^+^ bone marrow cell RNA sequencing studies, male C57BL/6 mice were subjected to three rounds of tail vein injections with EnzR EVs (10 µg EV protein per injection, 100 µl) or vehicle control (PBS) at 48 h intervals. One group of mice was also subjected to three rounds of HDL NP injections (100 µM, 100 µl) via tail vein on alternating days from EnzR EV injections, beginning 24 h prior to the first EnzR EV injection. 24 h after the final injection, mice were euthanized and bone marrow cells were isolated as described above. CD11b^+^ cells were then isolated from the bone marrow population using a commercially available separation kit (EasySep) according to the manufacturer's instructions. mRNA was then isolated using RNeasy isolation kit (Qiagen). Experiments were performed using *n* = 3 mice per group for CD11b^+^ RNA isolation and sequencing.

### 2.20 RNA sequencing of CD11b^+^ mouse bone marrow cells

Stranded total RNA‐seq was conducted in the Northwestern University NUSeq Core Facility. Briefly, total RNA examples were checked for quality on Agilent Bioanalyzer 2100 and quantified with Qubit fluorometer. The Illumina TruSeq Stranded Total RNA Library Preparation Kit was used to prepare sequencing libraries from 200 ng of total RNA samples. The Kit procedure was performed without modifications. This procedure includes rRNA depletion, remaining RNA purification and fragmentation, cDNA synthesis, 3′ end adenylation, Illumina adapter ligation, library PCR amplification and validation. lllumina NextSeq 500 Sequencer was used to sequence the libraries with the production of single‐end, 75 bp reads.

The quality of DNA reads, in FASTQ format, was evaluated using FastQC.  Adapters were trimmed, and reads of poor quality or aligning to rRNA sequences were filtered. The cleaned reads were aligned to the *Mus musculus* genome (mm10) using STAR (Dobin et al, 2013). Read counts for each gene were calculated using htseq‐count (Anders et al, 2015) in conjunction with a gene annotation file for mm10 obtained from UCSC (University of California Santa Cruz; http://genome.ucsc.edu). Normalization and differential expression were determined using DESeq2 (Love et al, 2014). The cutoff for determining significantly differentially expressed genes was an FDR‐adjusted *P*‐value less than 0.05. A pathway analysis was performed on both gene lists using GeneCoDis (Tabas‐Madrid et al, 2012; Nogales‐Cadenas et al, 2009; Carmona‐Saez et al, 2007) to identify pathways enriched with genes that are upregulated and downregulated. Results were obtained from *n* = 3 mice per group. RNA sequencing data has been uploaded to NCBI GEO database (Accession: GSE158012).

### 2.21 Western blot

For Western blotting, 20 µg or 5 µg of total protein were used for cell lysates and EV protein respectively. Samples were diluted in Laemmli buffer and incubated at 98°C for 5 min. Protein samples were resolved using Tris/Glycine/SDS pre‐cast polyacrylamide gels and a BioRad Western blot system running at 200 V for 32 min. Protein was transferred from polyacrylamide gel to polyvinylidene fluoride membranes via wet transfer for 90 min at 70 V. Membranes were then blocked in 5% non‐fat milk in TBS‐Tween (0.1% Tween 20) for 1 h at RT. Membranes were incubated with primary antibodies at dilutions recommended by the manufacturer (anti‐CD9, Cell Signaling Technology, Cat#: D8O1A, 1:500 dilution; anti‐CD63, Novus Biologicals, Cat#: NB100‐77913, 1:250 dilution; anti‐flotillin‐1, BD Biosciences, Cat#: 610820, 1:500 dilution; anti‐GM130, BD Biosciences, Cat# 610822, 1:500 dilution) in blocking buffer for either 3 h at RT or overnight at 4°C, washed three times in TBS‐Tween for 10 min, and incubated with HRP‐conjugated secondary antibodies in blocking buffer for 1 h at RT. Membranes were then washed three times in TBS‐Tween for 10 min and developed in ECL detection reagents (GE Healthcare).

### 2.22 Statistics and reproducibility

All statistical analyses were performed using GraphPad Prism. Statistical significance was calculated using either unpaired two‐tailed Student's *t*‐test or one‐way ANOVA with Tukey's post‐hoc test, two‐sided. *P < 0.05, **P < 0.01, ***P < 0.001. No statistical methods were used to pre‐determine sample sizes, blinding, or randomization methods. The precise *n* for all experiments is explicitly indicated in each relevant Materials and Methods section, as well as in the main text and figure captions for select experiments. Details regarding the use of technical and biological replicates are described in the Materials and Methods.

## 3 RESULTS AND DISCUSSION

**FIGURE 1:**
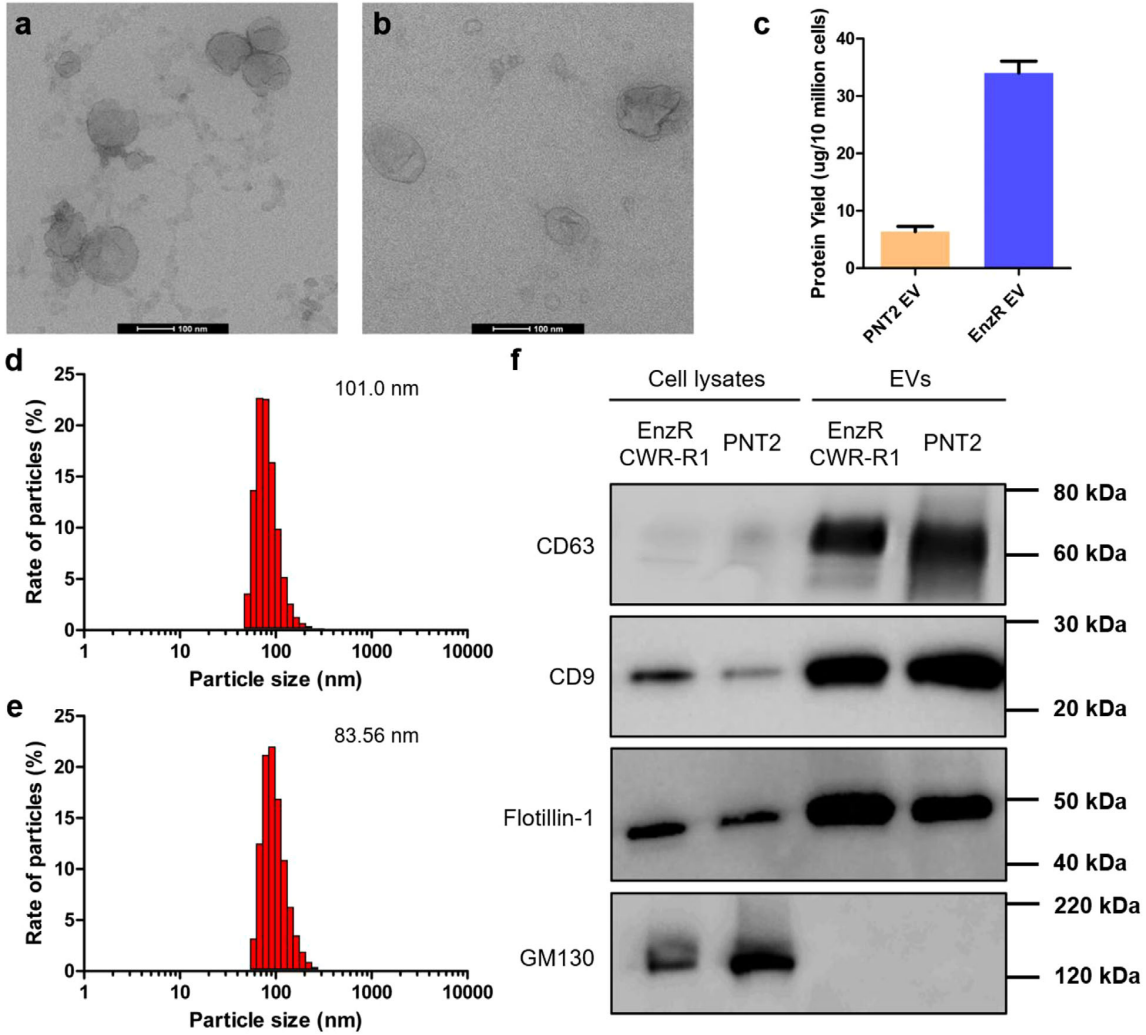
Characterization of EnzR and PNT2 EVs. Transmission electron microscopy imaging of (a) EnzR EVs and (b) PNT2 EVs. (c) Quantification of EV production by enzalutamide resistant CWR‐R1 cells (EnzR) and normal prostate epithelial cells (PNT2). Size distribution profiles of (d) EnzR EVs and (e) PNT2 EVs measured by dynamic light scattering. (f) Western blots of EnzR and PNT2 EVs and cell lysates for EV‐enriched proteins (CD63, CD9, and Flotillin‐1) and the non‐EV, *cis*‐Golgi protein GM130

We first characterized a previously unreported axis of intercellular communication between PCa cells and bone marrow myeloid cells. We selected enzalutamide resistant (EnzR) CWR‐R1 cells as a source of EVs (EnzR EVs) and as a model of bone metastatic PCa. These cells exhibit two important characteristics of late stage PCa in humans: 1) resistance to anti‐androgen therapy, and 2) a propensity to seed clinically relevant sites of PCa metastasis (e.g., lung, bone, and liver) when systemically injected into mice (Kregel et al., 2016). EVs from normal prostate epithelial cells (PNT2 EVs) were used as a control. EnzR and PNT2 EVs were characterized by multiple complementary techniques in accordance with the most recent guidelines for characterization of EVs (Thery et al., 2018). The size and morphology of EnzR and PNT2 EVs were evaluated using transmission electron microscopy (TEM) and dynamic light scattering (DLS). TEM revealed vesicles exhibiting a cup‐shaped morphology typical of sEVs (Figure 1a and b), while DLS demonstrated hydrodynamic diameters in the size regime of sEVs (EnzR: *D_H_* = 101.0 ± 23.0 nm, PNT2: *D_H_* = 83.56 ± 31 nm) (Figure 1d and e). Both EnzR and PNT2 EVs expressed EV proteins (CD63, CD9, and Flotillin‐1), while they importantly lacked the *cis*‐Golgi marker GM130 (Figure 1f), indicating EV isolates of high purity. Moreover, we found that EnzR CWR‐R1 cells produced approximately five‐fold more EVs (EV protein per cell) than PNT2 cells (Figure 1c).

**FIGURE 2:**
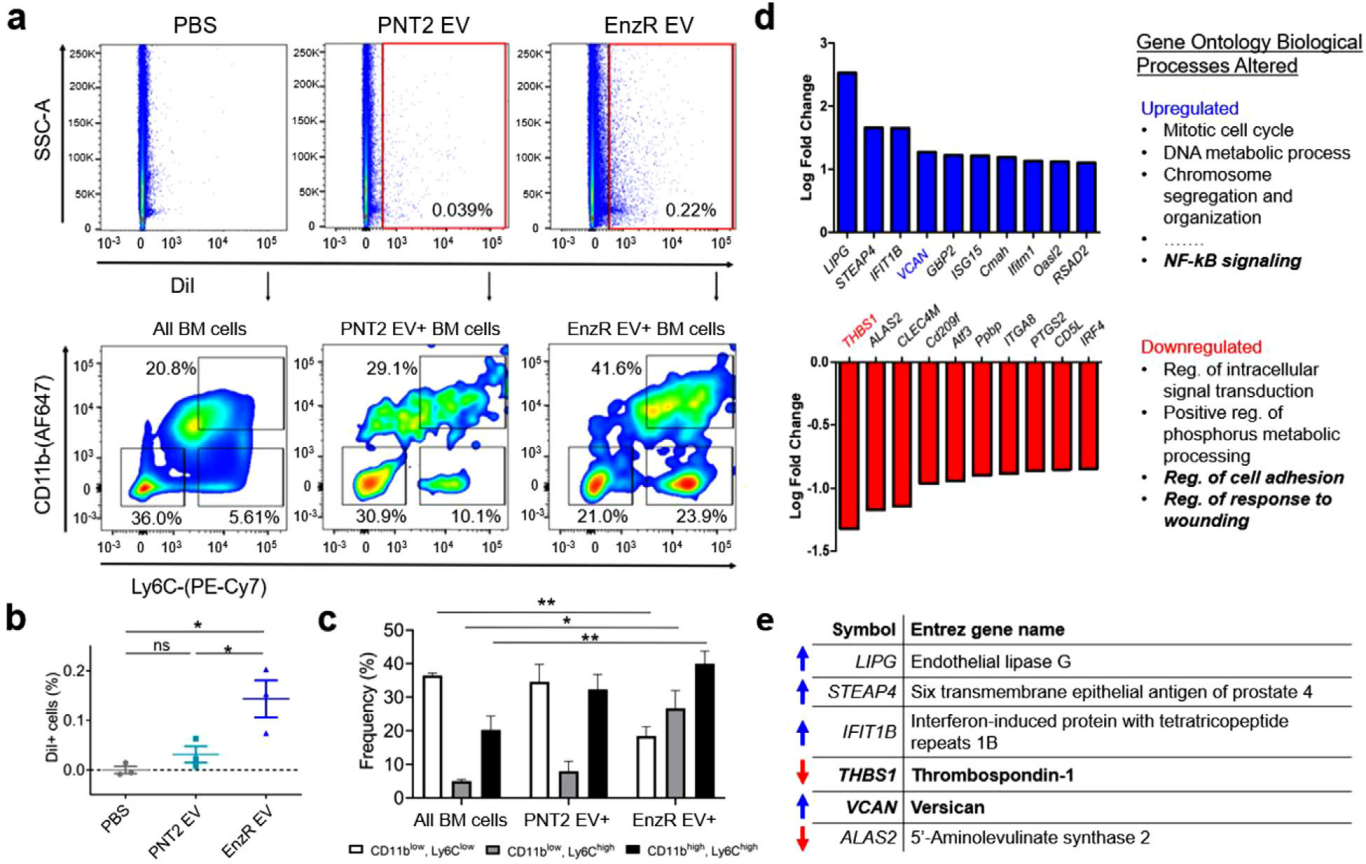
EnzR EVs mediate intercellular communication with bone marrow myeloid cells in vivo. (a) In vivo uptake of DiI‐labelled EnzR and PNT2 EVs in mouse bone marrow (top panel). Bottom panel displays the distribution of CD11b and Ly6C expressing cells. (b) Quantification of in vivo uptake of EnzR and PNT2 EVs in mouse bone marrow. (c) Distribution of CD11b and Ly6C expressing sub‐populations in EV‐targeted cells vs. PBS‐treated control total bone marrow. (d) Alterations in gene expression of CD11b^+^ bone marrow cells harvested from mice conditioned with EnzR EVs, determined by RNA sequencing (*n* = 3 per group), in relation to PBS treated control. (e) Entrez gene names for the genes with the greatest log2 fold changes in response to conditioning with EnzR EVs. One‐way ANOVA with Tukey's method, two‐sided was used to determine significance. *P < 0.05, **P < 0.01. Data are mean ± s.e.m

The site where circulating tumour cells initially seed the bone, whether in the appendicular or axial skeleton, is the highly vascular bone marrow compartment (Alix‐Panabieres, Riethdorf, & Pantel, 2008; Bidard et al., 2007; Pantel, 2010). Therefore, we first investigated whether EnzR EVs were efficiently taken up by bone marrow cells in vitro and in vivo. We found that fluorescently labelled (DiI) EnzR EVs were robustly uptaken by primary cultures of mouse bone marrow macrophages (BMMs) (Figure S1a) and by mouse bone marrow‐resident cells in vivo (Figure 2a and b) as determined by immunocytochemistry and flow cytometry, respectively. Notably, EnzR EVs were taken up by bone marrow cells in vivo to a greater extent than PNT2 EVs by approximately five‐fold (Figure 2b) (mean frequency of EV^+^ cells: PNT2 = 0.031%, EnzR = 0.14%).

Next, we defined the target cell populations of EnzR EVs in the bone marrow compartment with greater specificity. The population of EV‐targeted cells was interrogated for myeloid, monocytic, and granulocytic markers (CD11b, Ly6C, Ly6G, respectively). We found that the EnzR EV‐positive cell population was enriched in CD11b^high^, Ly6C^high^ cells (monocytes) (40.0%) compared to the baseline frequency in total bone marrow (20.3%) (Figure 2a and c). By contrast, there was no difference in the frequency of Ly6G expressing cells (Figure S1b) between EnzR EV‐targeted cells and total bone marrow cells, indicating preferential communication of EnzR EVs with monocytic over granulocytic BMDCs. Interestingly, the population of cells targeted by EnzR EVs was enriched in both CD11b^low^, Ly6C^high^ and CD11b^high^, Ly6C^high^ cells (Figure 2c). Both of these cell types have been recognized as osteoclast precursor populations (Jacome‐Galarza, Lee, Lorenzo, & Aguila, 2013; Takegahara et al., 2016), which led us to further investigate the impact of EnzR EVs on osteoclast differentiation as a feature of PMN formation later in the study.

**FIGURE 3:**
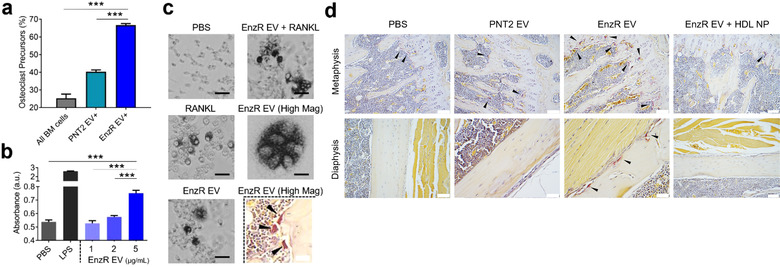
EnzR EVs stimulate NF‐κB signalling and promote osteoclast differentiation. (a) Quantification of the frequency of EV‐targeted cells in mouse bone marrow that are potential osteoclast precursors (CD11b^high^, Ly6C^high^ or CD11b^low^, Ly6C^high^) (*n* = 3 per group). Osteoclast precursor % reflects the percent of DiI positive bone marrow cells (EV treated) or total (PBS treated) bone marrow cells that were osteoclast precursors. (b) NF‐κB activity assay using THP1‐Dual monocytes treated with varying concentrations of EnzR EVs (*n* = 4 per group). Absorbance reflects secreted alkaline phosphatase (SEAP) activity which is indicative of NF‐κB activation. (c) In vitro osteoclast differentiation assay using RAW264.7 osteoclast precursor cells. TRAP+ (dark staining), multinucleated cells are osteoclasts. Scale bars are 50 µm. (d) TRAP staining of hind limb bones. Arrowheads indicate osteoclasts (TRAP+ multi‐nucleated cells, inside or outside lacunae). Scale bars are 200 µm. One‐way ANOVA with Tukey's method, two‐sided was used to determine significance. ***P < 0.001. Data are mean ± s.e.m

Next, we performed RNA sequencing of target bone marrow myeloid cells (CD11b^+^) to identify alterations in gene expression induced by EnzR EVs. To do this, we simulated PMN formation in vivo according to precedent literature (Costa‐Silva et al., 2015; Peinado et al., 2012; Plebanek et al., 2017) by subjecting mice to three rounds of systemic injections of EnzR EVs, a process we define as “conditioning”. The most significantly upregulated and downregulated genes by EnzR EV conditioning are displayed in Figure 2d. Two of the altered genes were of particular interest with respect to PMN formation: thrombospondin‐1 (TSP1; *THBS1*) was the most significantly downregulated gene, while the chondroitin sulfate proteoglycan, versican (VCAN), was significantly upregulated. Interestingly, TSP1 and VCAN have each been reported to play significant roles at the lung PMN; and in both cases, myeloid cells were responsible for aberrant TSP1 or VCAN expression (Catena et al., 2013; Gao et al., 2012). To our knowledge, neither of these alterations has been reported at the bone PMN, or in PCa bone metastases. Using Gene Ontology clustering analysis, we observed that the most upregulated biological processes were related to cell division and proliferation (Figure 2d), while NF‐κB activity was also upregulated. Activation of NF‐κB signalling was later confirmed using an in vitro reporter system (Figure 3b). Among the most significantly downregulated processes were regulation of cell adhesion and response to wounding, both of which are relevant to PMN formation.

Because EnzR EVs targeted osteoclast precursor populations in vivo (Figures 2c and 3a), we hypothesized that EnzR EVs may promote osteoclast differentiation as a feature of bone PMN formation. In the setting of bone metastasis, increased osteoclast and osteoblast activity is favourable for tumour cells due to the constitutive release of nutrients and growth factors caused by rapid bone formation and resorption (Shiozawa, Eber, Berry, & Taichman, 2015; Weidle, Birzele, Kollmorgen, & Ruger, 2016; Zheng, Li, & Kang, 2016). Elevated osteoclast activity in particular is associated with increased burden of bone metastases in PCa (Hirata et al., 2016; Keller & Brown, 2004; Roato et al., 2008; Sottnik & Keller, 2013). Moreover, an inhibitor of the osteoclast activating protein, receptor activator of NF‐ κB ligand (RANKL), delayed the development of bone metastasis among patients with high risk PCa (Smith et al., 2012). To test the impact of EnzR EVs on osteoclast activity, we first used a well‐established in vitro model system (RAW264.7 cells) for osteoclast differentiation (Collin‐Osdoby & Osdoby, 2012). We found that EnzR EVs promoted osteoclast differentiation in vitro with and without the addition of RANKL (Figure 3c). Confirming these findings in vivo, we observed that osteoclasts were more abundant in the hind limbs of mice conditioned with EnzR EVs compared to vehicle (PBS) control, while PNT2 EVs had no impact on osteoclast differentiation (Figure 3d) (Figure S2a). We also found that EnzR EVs stimulated NF‐κB activity (a critical intracellular signalling pathway for osteoclast differentiation) in human monocytes in a dose‐dependent manner (Figure 3b). We hypothesized that EnzR EVs may express RANK ligand (RANKL) as a means to stimulate osteoclast differentiation, however Western blot revealed no RANKL expression in EnzR EVs, in contrast with positive control PC3 EVs (Figure S2b).

Recent evidence suggests that EV‐mediated intercellular communication may be influenced by cholesterol homeostasis in recipient cells. For instance, treating target cells with the non‐specific cholesterol sequestrant, methyl‐β‐cyclodextrin (MβCD), can reduce EV uptake by disrupting lipid rafts at the cell surface (Svensson et al., 2013). As a result, we hypothesized that PCa EV‐mediated signalling with bone marrow cells may be inhibited or abolished by reducing cholesterol in the target bone marrow cells. To thoroughly investigate this hypothesis in vivo, we required an agent that reduces cellular cholesterol in a manner similar to MβCD, while also being compatible with systemic delivery in animal models. In mammals, cholesterol transport is mediated by endogenous lipoproteins that circulate in the bloodstream. The lipoprotein sub‐species that is primarily responsible for reducing cellular cholesterol in vivo is high‐density lipoprotein (HDL) (Lund‐Katz & Phillips, 2010). Therefore, we employed a nanoparticle mimic of native HDL (HDL NP) (Figure S3) which we have previously characterized for in vitro inhibition of EV uptake (Plebanek et al., 2015), but which is also compatible with systemic delivery in vivo. HDL NPs inhibit EV uptake in a manner similar to MβCD by reducing cellular cholesterol and disrupting lipid raft stability (Plebanek et al., 2015). Importantly, HDL NPs specifically target cells expressing the high‐affinity receptor for HDL, scavenger receptor B‐1 (SR‐B1), including myeloid‐derived cells (Plebanek, Bhaumik, Bryce, & Thaxton, 2018), and have demonstrated no significant toxicities when used in animal models (Henrich & Thaxton, 2019; Rink et al., 2018; Yang et al., 2013). Therefore, we investigated the cholesterol dependence of EnzR EV‐mediated intercellular communication with myeloid cells using HDL NPs. The HDL NP synthesis scheme is depicted in Figure S3.

**FIGURE 4:**
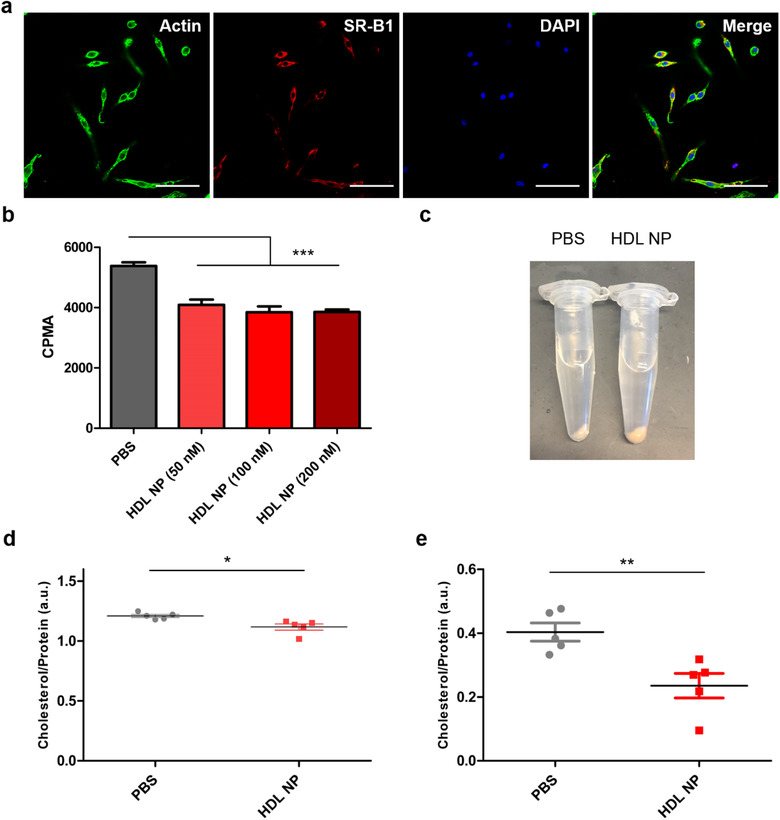
Bone marrow myeloid cells express SR‐B1 and are susceptible to HDL NP‐mediated reduction of cellular cholesterol in vitro and in vivo. (a) Immunocytochemistry of primary mouse BMM cultures to detect expression of the native HDL receptor (and target receptor of HDL NPs), SR‐B1. Scale bar = 50 µm. (b) Cellular cholesterol measurements of THP1 monocytes after treatment with HDL NPs or PBS in a radiolabel assay. (c) Bone marrow cell pellets harvested from mice after systemic injection of HDL NPs (100 µl, 1 µM) or PBS. Yellow/red discoloration is imparted by the gold nanoparticle core. (d) Quantification of cellular cholesterol in total bone marrow cells after systemic injection with HDL NPs or PBS. (e) Quantification of cellular cholesterol in bone marrow monocytes (EasySep Isolation Kit) after systemic injection with HDL NPs or PBS. One‐way ANOVA with Tukey's method, two‐sided (b) and two‐sided Welch's *t*‐test (d,e) were used to determine significance. *P < 0.05, **P < 0.01, ***P < 0.001. Data are mean ± s.e.m

We first confirmed that mouse bone marrow myeloid cells expressed SR‐B1 and would therefore be susceptible to HDL NP‐mediated reduction of cellular cholesterol (Figure 4a). Next, we demonstrated that HDL NP treatment reduced cellular cholesterol in myeloid‐derived cells using human monocytes in an in vitro radiolabel assay (Figure 4b). We then confirmed that HDL NPs reduced myeloid cellular cholesterol in vivo by treating mice with HDL NPs prior to harvesting bone marrow cells and quantifying cellular cholesterol. Systemically injected HDL NPs entered the bone marrow compartment and reduced cellular cholesterol in the total bone marrow cell population (Figure 4c and d). Importantly, we observed a pronounced reduction of cellular cholesterol in bone marrow monocytes specifically (Figure 4e), the dominant target cell population of EnzR EVs.

**FIGURE 5:**
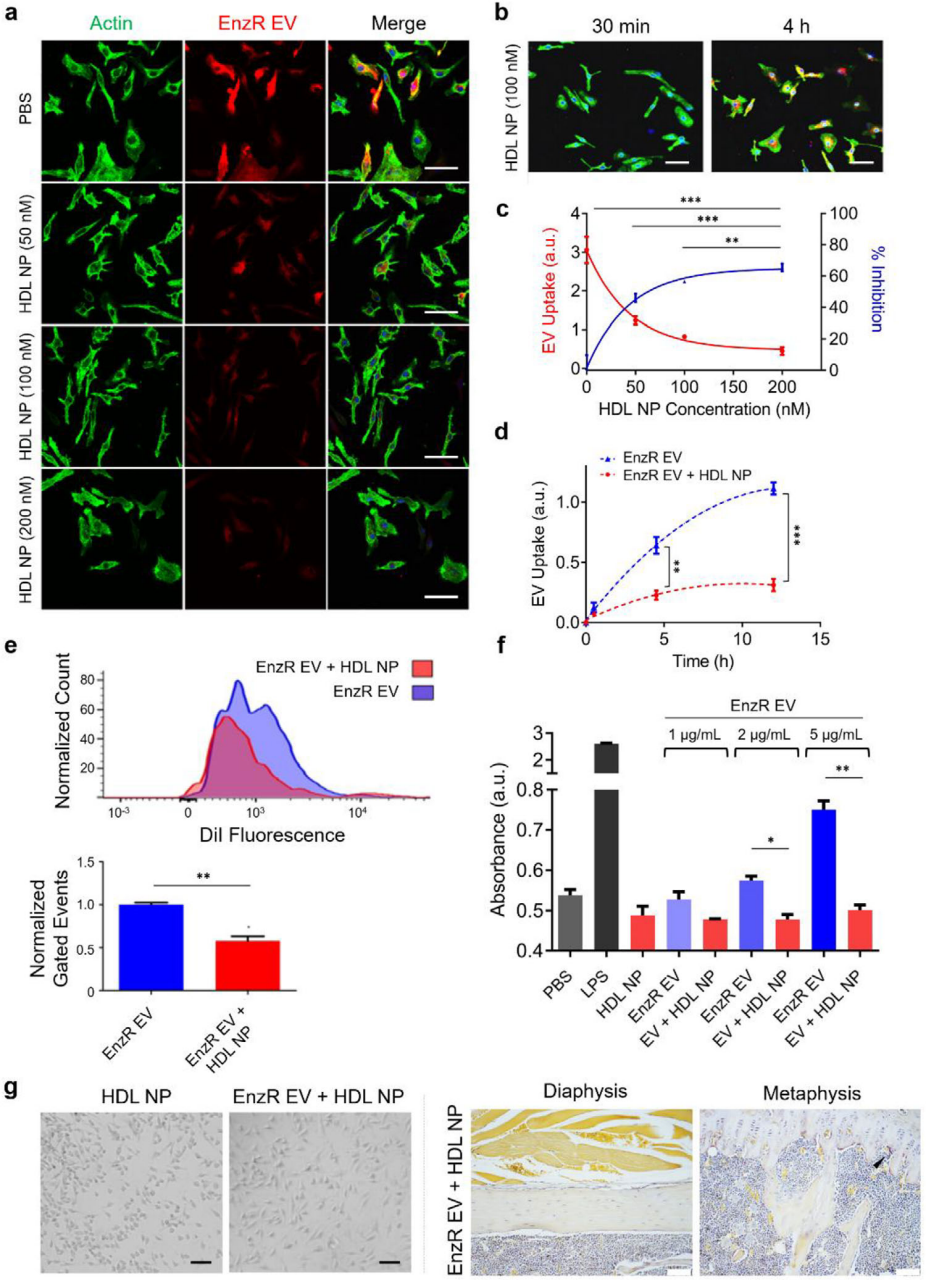
EnzR EV‐mediated intercellular communication with bone marrow myeloid cells is inhibited by reduction of myeloid cellular cholesterol. (a) In vitro uptake of DiI‐labelled EnzR EVs (red) in murine BMMs pre‐treated with HDL NPs at varying concentrations. Green: actin. Blue: DAPI. Scale bars are 50 µm. (b) In vitro uptake of DiI‐labelled EnzR EVs (red) at two different time points in BMMs pre‐treated with HDL NPs. Scale bars are 50 µm. (c) Dose and (d) time dependence of HDL NP‐mediated inhibition of EnzR EV uptake. (e) In vivo uptake of DiI‐labelled EnzR EVs in total mouse bone marrow with and without HDL NP pre‐treatment, via flow cytometry. (f) NF‐κB activity in human monocytes treated with EnzR EVs with and without HDL NP pre‐treatment. (g) TRAP staining of RAW264.1 cells (left panel) or mouse hind limb sections (right panel) after conditioning with EnzR EVs and pre‐treatment with HDL NPs. Scale bars are 50 µm (left panel) and 200 µm (right panel). One‐way ANOVA with Tukey's method, two‐sided (c,d,f) and two‐tailed Welch's *t*‐test (e) were used to determine significance. *P < 0.05, **P < 0.01, ***P < 0.001. Data are mean ± s.e.m

We then investigated whether HDL NP treatment inhibited EnzR EV‐mediated intercellular communication with myeloid cells in vitro and in vivo. We first found that HDL NPs inhibited EnzR EV uptake by BMMs in a time and dose‐dependent fashion (Figure 5a–d). Next, we observed that systemic administration of HDL NPs reduced the uptake of EnzR EVs by bone marrow‐resident cells in vivo via flow cytometry (Figure 5e). Importantly, to demonstrate target specificity, we found that HDL NP treatment had no impact on EnzR EV uptake in BMM cultures from SR‐B1^–/‐^ mice (Figure S4), indicating that HDL NP‐mediated inhibition requires SR‐B1. To determine whether the cholesterol dependence of PCa EV uptake in BMMs was restricted to EVs derived from EnzR CWR‐R1 cells, in vitro uptake experiments were repeated using PCa EVs derived from DU145, PC3, LNCaP, EnzR LNCaP, and CWR‐R1 (non‐resistant) cells. HDL NPs were found to significantly inhibit the uptake of each of these PCa EV populations in BMMs (Figure S5), strongly suggesting that the cholesterol dependence of PCa EV communication with bone marrow‐resident cells is a general phenomenon.

Next, we determined the impact of HDL NPs on EnzR EV function. We found that HDL NPs inhibited EnzR EV‐mediated NF‐κB signalling (Figure 5f) and abolished EnzR EV‐induced osteoclast differentiation in vitro and in vivo (Figure 5 g) (Figure S2). Importantly, we also found that HDL NP pre‐treatment prevented EnzR EV‐mediated reduction of TSP1 gene expression in bone marrow myeloid cells in vivo (Figure S6a). Interestingly, HDL NP pre‐treatment did not impact the increased expression of VCAN caused by EnzR EVs (Figure S6b).

**FIGURE 6:**
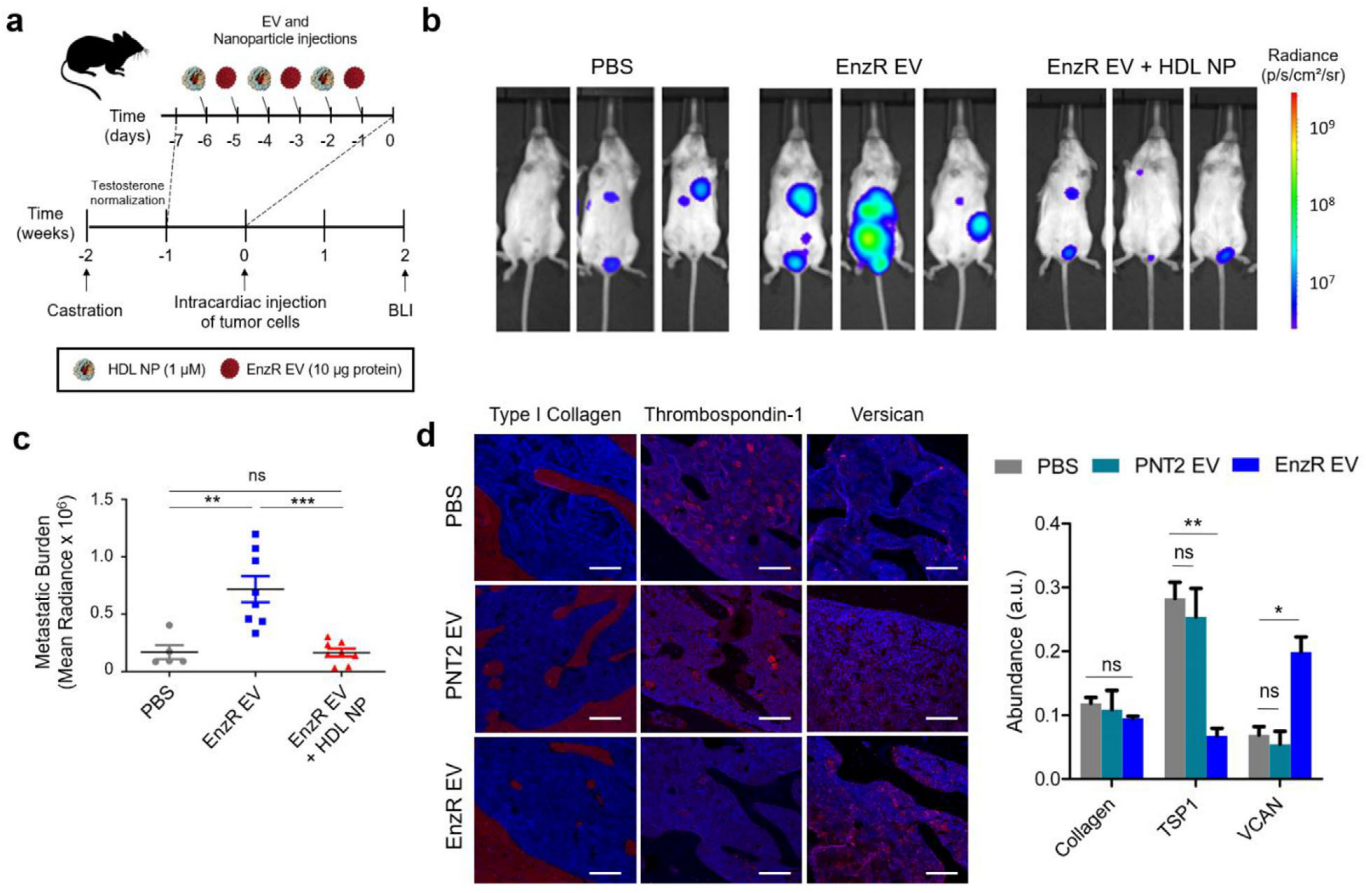
EnzR EVs enhance metastasis in a mouse model of metastatic PCa in a cholesterol‐dependent manner, and alter the composition of the bone marrow ECM. (a) Timeline for the metastatic PCa mouse model, with EnzR EV and HDL NP injection scheme. BLI = bioluminescence imaging. (b) Metastatic PCa tumour burden at two weeks post‐intracardiac injection of tumour cells via BLI. (c) Quantification of metastatic tumour burden at two weeks for mice treated with PBS (*n* = 5), EnzR Exo (*n* = 8), or EnzR Exo + HDL NP (*n* = 8). (d) Immunohistochemistry of hind limb bone tissue from mice treated with EnzR EVs or vehicle (PBS) control (left panel), and ECM quantification (right panel). Scale bars are 200 µm. One‐way ANOVA with Tukey's method, two‐sided was used to determine significance. *P < 0.05, **P < 0.01, ***P < 0.001. Data are mean ± s.e.m

Using a metastatic PCa mouse model which has been shown to establish tumours at clinically relevant sites of PCa metastasis [29], we then tested whether EnzR EV conditioning promoted metastatic tumour burden. A timeline of the model, injection scheme, and monitoring with bioluminescence imaging is shown in Figure 6a. To specifically determine whether EnzR EV conditioning altered the initial seeding and growth of tumour cells, we quantified metastatic tumour burden at two weeks after intracardiac injection of tumour cells. We found that EnzR EV conditioning significantly enhanced metastatic tumour burden (Figure 6b). Strikingly, mice that were pre‐treated with HDL NPs in addition to EnzR EV conditioning exhibited no enhancement of metastatic tumour burden (Figure 6b and c).

**FIGURE 7:**
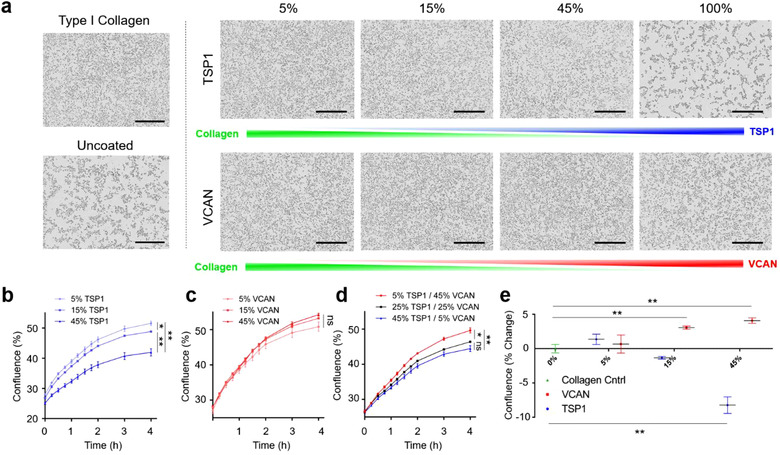
Reduced TSP1 and elevated VCAN are favourable for PCa tumour cell adhesion to the ECM. (a) Live cell imaging of EnzR CWR‐R1 cells seeded onto ECM substrates varying in Type I collagen, TSP1, and VCAN. Snapshot images shown were taken 4 h after seeding. Scale bars are 400 µm. (b) Quantification of EnzR CWR‐R1 cell adhesion to collagenous ECM substrates with variable TSP1 composition, (c) varying VCAN composition, and (d) co‐varying TSP1 and VCAN composition. (e) Aggregate analysis at 4 h post‐seeding of changes in cellular adhesion of EnzR CWR‐R1 cells to substrates with univariate changes in TSP1 and VCAN composition. One‐way ANOVA with Tukey's method, two‐sided was used to determine significance. *P < 0.05, **P < 0.01. Data are mean ± s.e.m

We then sought to shed additional light on the mechanism by which EnzR EV conditioning led to enhanced metastasis. Motivated by the RNA sequencing results in Figure 2d, we proceeded to determine whether pro‐tumorigenic alterations in the mRNA expression of TSP1 and VCAN in CD11b^+^ cells were reflected in the protein content of the bone marrow niche in general. Immunohistochemistry revealed that EnzR EV‐conditioned mice exhibited reduced TSP1 and increased VCAN expression in the bone marrow compartment (Figure 6d), consistent with RNA sequencing results. Type I collagen, by contrast, remained stable with EnzR EV conditioning. Moreover, PNT2 EVs did not significantly impact TSP1 or VCAN expression (Figure 6d). We hypothesized that EnzR EV‐induced alterations in TSP1 and VCAN expression may enhance the ability of tumour cells to seed the metastatic microenvironment by increasing stable cellular adhesion to the ECM. To test this, we conducted an in vitro cellular adhesion assay in which the ECM composition of two‐dimensionally coated tissue culture wells was varied, using Type I collagen as a filler material. Live cell imaging was used to determine the efficiency with which EnzR CWR‐R1 cells adhered and spread to each substrate. Confluence curves exhibited consistent trends across samples, enabling us to identify distinct adhesion and proliferation phases, with the adhesion phase taking place during the first 4 h (Figure S7). Consistent with our hypothesis, we found that greater proportions of TSP1 in ECM coatings led to diminished adhesion of EnzR CWR‐R1 cells, while greater proportions of VCAN increased adhesion (Figure 7a–e). While there were no statistical differences between groups with 5, 15, and 45% VCAN substrates, there was a significant increase in cellular adhesion between 15 and 45% VCAN compared to collagen only control (Figure 7c). The trends observed for TSP1 and VCAN were consistent when each protein was varied in isolation, and when the two proteins were co‐varied in the same substrates (Figure 7d). Our findings are consistent with previous reports that TSP1 can function as an anti‐adhesive matricellular protein in other settings (Murphy‐Ullrich & Hook, 1989; Murphy‐Ullrich et al., 1991).

**FIGURE 8:**
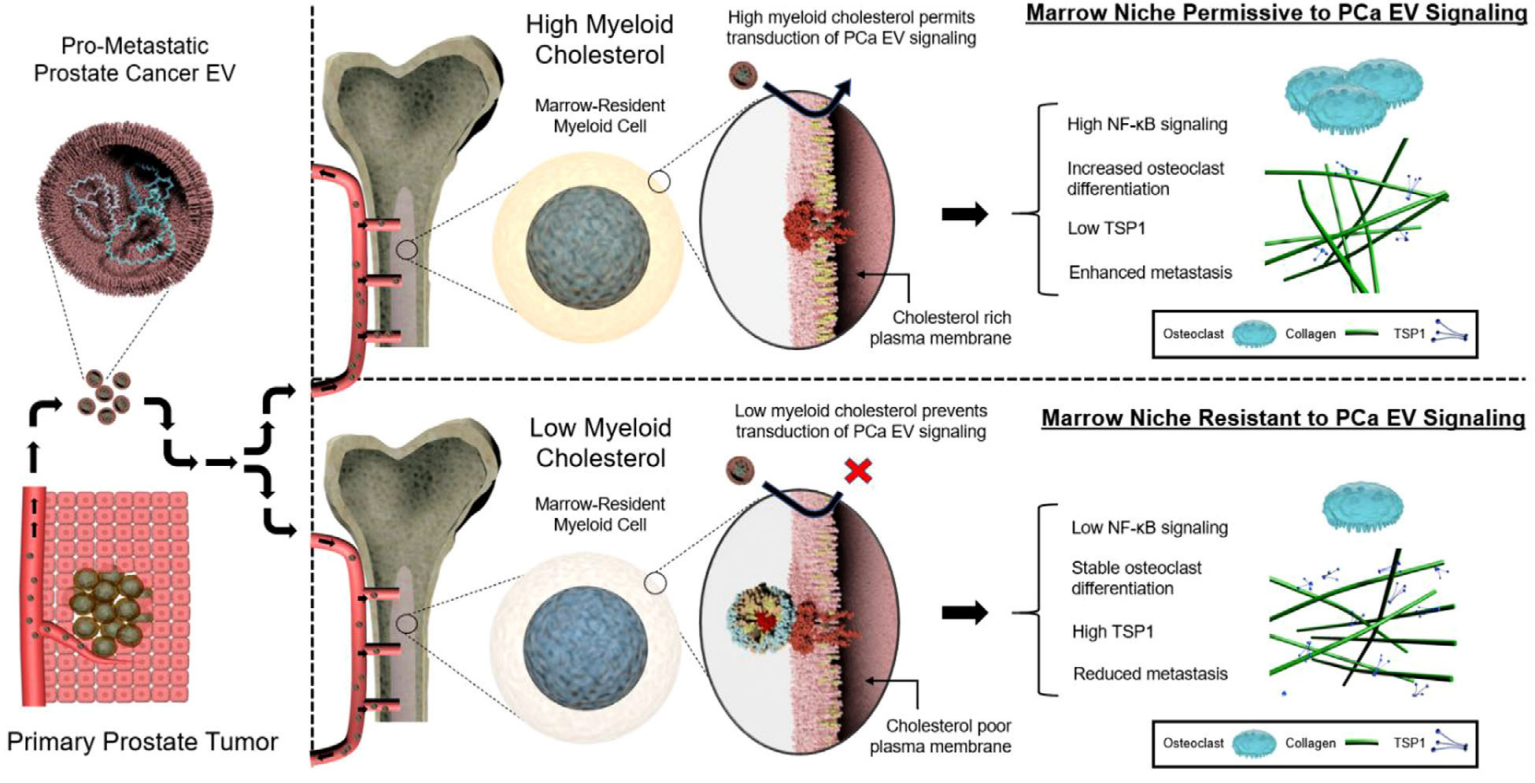
Graphical abstract illustrating the cholesterol‐dependence of PCa EV communication with bone marrow cells and metastasis. Top right panel depicts PCa EV communication with bone marrow myeloid cells with no cholesterol‐modifying intervention, in which bone marrow‐resident myeloid cells are rich in cholesterol. Bottom right panel depicts PCa EV communication with bone marrow cells after HDL NP treatment, in which the cholesterol content of bone marrow‐resident myeloid cells is reduced

A graphical abstract summarizing the cholesterol‐dependence of PCa EV‐mediated signalling in the bone marrow compartment is displayed in Figure 8.

## 4 CONCLUSIONS

Over the past two decades, Stephen Paget's prescient 19^th^ century “seed and soil” hypothesis has now developed into a diverse literature on PMN formation, a significant portion of which attests to the important role that EVs play in promoting metastasis. However, modifiable factors intrinsic to pre‐metastatic sites that render them more or less permissive to pro‐metastatic EV signalling have not been identified. Our results show that a crucial PMN target tissue, bone marrow, can be rendered resistant to the transduction of PCa EV signalling by reducing myeloid cell cholesterol. Consistent with these findings, metabolomic studies have previously demonstrated that bone metastases are heavily laden with cholesterol compared to healthy bone, and PCa bone tumours in particular bear especially high cholesterol burdens (127.3 vs. 35.85 mg/g tissue) (Thysell et al., 2010). Further, elevated serum total cholesterol and reduced HDL cholesterol are both associated with progression of locally confined PCa to metastatic PCa (Mondul, Clipp, Helzlsouer, & Platz, 2010; Platz et al., 2009; Platz, Clinton, & Giovannucci, 2008; Van Hemelrijck et al., 2011), while statin use is also associated with reduced incidence of advanced disease (Jacobs et al., 2007). Our results provide evidence for a PMN‐based mechanism that could partly explain these clinical and epidemiological observations.

Aberrant cholesterol homeostasis can influence cancer progression and metastasis by perturbing neoplastic cells directly (Alfaqih et al., 2017; Gallagher et al., 2017; Jiang et al., 2019). However, it perhaps shouldn't be surprising that cholesterol, and likely other metabolic factors, also play critical gate‐keeping roles with respect to the reception of pro‐metastatic signals by target cells at distant sites, which we demonstrate here. We anticipate that these results will motivate strategic diagnostic and therapeutic interventions aimed at preventing metastasis by modulating global, site‐specific, or cellular metabolic factors that regulate PMN formation. More generally, these results contribute to our understanding of how intercellular communication is deciphered by recipient cells according to their cholesterol metabolic state.

## CONFLICT OF INTEREST

The authors declare no competing interests.

## AUTHOR CONTRIBUTIONS

The study was conceived by Stephen E. Henrich and C. Shad Thaxton. Stephen E. Henrich designed, performed, and analyzed results all for experiments, assisted by Kaylin M. McMahon, Michael P. Plebanek, Andrea E. Calvert, Timothy J. Feliciano, Sam Parrish, Fabio Tavora, Anthony Mega and Anthony Mega. C. Shad Thaxton and Benedito A. Carneiro supervised all research, participated in the interpretation of results, and edited the manuscript.

## Supporting information



Supporting Information

## Data Availability

RNA sequencing data has been uploaded to NCBI GEO database (Accession: GSE158012) and will be publicly available on October 1^st^, 2021. All other data supporting the findings of this study can be found in the article and supplementary materials.

## References

[jev212042-bib-0001] Alfaqih, M. A. , Nelson, E. R. , Liu, W. , Safi, R. , Jasper, J. S. , Macias, E. , … Freedland, S. J. (2017). CYP27A1 Loss dysregulates cholesterol homeostasis in prostate cancer. Cancer Research, 77(7), 1662–1673. 28130224 10.1158/0008-5472.CAN-16-2738 PMC5687884

[jev212042-bib-0002] Alix‐Panabieres, C. , Riethdorf, S. , & Pantel, K. (2008). Circulating tumor cells and bone marrow micrometastasis. Clinical Cancer Research, 14(16), 5013–5021. 18698019 10.1158/1078-0432.CCR-07-5125

[jev212042-bib-0003] Bethani, I. , Skanland, S. S. , Dikic, I. , & Acker‐Palmer, A. (2010). Spatial organization of transmembrane receptor signalling. Embo Journal, 29(16), 2677–2688. 10.1038/emboj.2010.175 PMC2924650 20717138

[jev212042-bib-0004] Bidard, F. C. , Vincent‐Salomon, A. , Sastre, X. , Sigal‐Zafrani, A. , Nos, C. , Mignot, L. , … Pierga, J. Y. (2007). Bone marrow micrometastasis and circulating tumor cells are respectively strong prognostic factors in early and metastatic breast cancer, a comparative study on 759 patients. Breast Cancer Research and Treatment, 106, S26–S26.

[jev212042-bib-0005] Bubendorf, L. , Schopfer, A. , Wagner, U. , Sauter, G. , Moch, H. , Willi, N. , … Mihatsch, M. J. (2000). Metastatic patterns of prostate cancer: an autopsy study of 1,589 patients. Human Pathology, 31(5), 578–583. 10836297 10.1053/hp.2000.6698

[jev212042-bib-0006] Catena, R. , Bhattacharya, N. , El Rayes, T. , Wang, S. M. , Choi, H. , Gao, D. C. , … Mittal, V. (2013). Bone marrow‐derived Gr1(+) cells can generate a metastasis‐resistant microenvironment via induced secretion of thrombospondin‐1. Cancer Discovery, 3(5), 578–589. 23633432 10.1158/2159-8290.CD-12-0476 PMC3672408

[jev212042-bib-0007] Chow, A. , Zhou, W. Y. , Liu, L. , Fong, M. Y. , Champer, J. , Van Haute, D. , … Wang, S. E. (2014). Macrophage immunomodulation by breast cancer‐derived exosomes requires toll‐like receptor 2‐mediated activation of NF‐kappa B. Scientific Reports, 4, 5750. 25034888 10.1038/srep05750 PMC4102923

[jev212042-bib-0008] Collin‐Osdoby, P. , & Osdoby, P. (2012). RANKL‐mediated osteoclast formation from murine RAW 264.7 cells. Methods in Molecular Biology (Clifton, N.J.), 816, 187–202. 10.1007/978-1-61779-415-5_13 22130930

[jev212042-bib-0009] Costa‐Silva, B. , Aiello, N. M. , Ocean, A. J. , Singh, S. , Zhang, H. , Thakur, B. K. , … Lyden, D. (2015). Pancreatic cancer exosomes initiate pre‐metastatic niche formation in the liver. Nature Cell Biology, 17(6), 816–826. 25985394 10.1038/ncb3169 PMC5769922

[jev212042-bib-0010] Dai, J. , Escara‐Wilke, J. , Keller, J. M. , Jung, Y. , Taichman, R. S. , Pienta, K. J. , & Keller, E. T. (2019). Primary prostate cancer educates bone stroma through exosomal pyruvate kinase M2 to promote bone metastasis. Journal of Experimental Medicine, 216(12), 2883–2899. 10.1084/jem.20190158 PMC6888980 31548301

[jev212042-bib-0011] Epand, R. M. , Sayer, B. G. , & Epand, R. F. (2005). Caveolin scaffolding region and cholesterol‐rich domains in membranes. Journal of Molecular Biology, 345(2), 339–350. 15571726 10.1016/j.jmb.2004.10.064

[jev212042-bib-0012] Fessler, M. B. , & Parks, J. S. (2011). Intracellular lipid flux and membrane microdomains as organizing principles in inflammatory cell signaling. Journal of Immunology, 187(4), 1529–1535. 10.4049/jimmunol.1100253 PMC3151145 21810617

[jev212042-bib-0013] Gallagher, E. J. , Zelenko, Z. , Neel, B. A. , Antoniou, I. M. , Rajan, L. , Kase, N. , & LeRoith, D. (2017). Elevated tumor LDLR expression accelerates LDL cholesterol‐mediated breast cancer growth in mouse models of hyperlipidemia. Oncogene, 36(46), 6462–6471. 28759039 10.1038/onc.2017.247 PMC5690879

[jev212042-bib-0014] Gao, D. C. , Joshi, N. , Choi, H. J. , Ryu, S. H. , Hahn, M. , Catena, R. , … Mittal, V. (2012). Myeloid progenitor cells in the premetastatic lung promote metastases by inducing mesenchymal to epithelial transition. Cancer Research, 72(6), 1384–1394. 22282653 10.1158/0008-5472.CAN-11-2905 PMC8543151

[jev212042-bib-0015] Henrich, S. E. , & Thaxton, C. S. (2019). An update on synthetic high‐density lipoprotein‐like nanoparticles for cancer therapy. Expert Review of Anticancer Therapy, 19(6), 515–528. 31148521 10.1080/14737140.2019.1624529

[jev212042-bib-0016] Hirata, T. , Park, S. C. , Muldong, M. T. , Wu, C. N. , Yamaguchi, T. , Strasner, A. , & … Jamieson, C. A. M. (2016). Specific bone region localization of osteolytic versus osteoblastic lesions in a patient‐derived xenograft model of bone metastatic prostate cancer. Asian Journal of Urology, 3(4), 229–239. 29264191 10.1016/j.ajur.2016.09.001 PMC5730873

[jev212042-bib-0017] Hoop, C. L. , Sivanandam, V. N. , Kodali, R. , Srnec, M. N. , & van der Wel, P. C. A. (2012). Structural characterization of the caveolin scaffolding domain in association with cholesterol‐rich membranes. Biochemistry, 51(1), 90–99. 22142403 10.1021/bi201356v PMC3290515

[jev212042-bib-0018] Hoshino, A. , Costa‐Silva, B. , Shen, T.‐L. , Rodrigues, G. , Hashimoto, A. , Mark, M. T. , … Lyden, D. (2015). Tumour exosome integrins determine organotropic metastasis. Nature, 527(7578), 329–335. 26524530 10.1038/nature15756 PMC4788391

[jev212042-bib-0019] Jacobs, E. J. , Rodriguez, C. , Bain, E. B. , Wang, Y. , Thun, M. J. , & Calle, E. E. (2007). Cholesterol‐lowering drugs and advanced prostate cancer incidence in a large U.S. cohort. Cancer Epidemiology, Biomarkers & Prevention, 16(11), 2213–2217. 10.1158/1055-9965.EPI-07-0448 17971518

[jev212042-bib-0020] Jacome‐Galarza, C. E. , Lee, S. K. , Lorenzo, J. A. , & Aguila, H. L. (2013). Identification, characterization, and isolation of a common progenitor for osteoclasts, macrophages, and dendritic cells from murine bone marrow and periphery. Journal of Bone and Mineral Research: The Official Journal of the American Society for Bone and Mineral Research, 28(5), 1203–1213. 10.1002/jbmr.1822 PMC3625454 23165930

[jev212042-bib-0021] Jiang, S. , Wang, X. , Song, D. , Liu, X. , Gu, Y. , Xu, Z. , … Gao, S. (2019). Cholesterol induces epithelial‐to‐mesenchymal transition of prostate cancer cells by suppressing degradation of EGFR through APMAP. Cancer Research, 79(12), 3063–3075. 30987997 10.1158/0008-5472.CAN-18-3295

[jev212042-bib-0022] Karnezis, T. , Shayan, R. , Caesar, C. , Roufail, S. , Harris, N. C. , Ardipradja, K. , … Stacker, S. A. (2012). VEGF‐D promotes tumor metastasis by regulating prostaglandins produced by the collecting lymphatic endothelium. Cancer Cell, 21(2), 181–195. 22340592 10.1016/j.ccr.2011.12.026

[jev212042-bib-0023] Keller, E. T. , & Brown, J. (2004). Prostate cancer bone metastases promote both osteolytic and osteoblastic activity. Journal of Cellular Biochemistry, 91(4), 718–729. 14991763 10.1002/jcb.10662

[jev212042-bib-0024] Kregel, S. , Chen, J. L. , Tom, W. , Krishnan, V. , Kach, J. , Brechka, H. , … Griend, D. J. V. (2016). Acquired resistance to the second‐generation androgen receptor antagonist enzalutamide in castration‐resistant prostate cancer. Oncotarget, 7(18), 26259–26274. 27036029 10.18632/oncotarget.8456 PMC5041979

[jev212042-bib-0025] Lajoie, P. , & Nabi, I. R. (2010). Lipid rafts, caveolae, and their endocytosis. International Review of Cell and Molecular Biology, 282, 135–163. 20630468 10.1016/S1937-6448(10)82003-9

[jev212042-bib-0026] Liu, Y. , & Cao, X. (2016). Characteristics and significance of the pre‐metastatic niche. Cancer Cell, 30(5), 668–681. 27846389 10.1016/j.ccell.2016.09.011

[jev212042-bib-0027] Lobb, R. J. , Lima, L. G. , & Moller, A. (2017). Exosomes: key mediators of metastasis and pre‐metastatic niche formation. Seminars in Cell & Developmental Biology, 67, 3–10. 28077297 10.1016/j.semcdb.2017.01.004

[jev212042-bib-0028] Lund‐Katz, S. , & Phillips, M. C. (2010). High density lipoprotein structure‐function and role in reverse cholesterol transport. Sub‐Cellular Biochemistry, 51, 183–227. 20213545 10.1007/978-90-481-8622-8_7 PMC3215094

[jev212042-bib-0029] Maas, S. L. N. , Breakefield, X. O. , & Weaver, A. M. (2017). Extracellular vesicles: unique intercellular delivery vehicles. Trends in Cell Biology, 27(3), 172–188. 27979573 10.1016/j.tcb.2016.11.003 PMC5318253

[jev212042-bib-0030] Martinez‐Seara, H. , Rog, T. , Karttunen, M. , Vattulainen, I. , & Reigada, R. (2010). Cholesterol induces specific spatial and orientational order in cholesterol/phospholipid membranes. Plos One, 5(6), e11162. 20567600 10.1371/journal.pone.0011162 PMC2887443

[jev212042-bib-0031] Mondul, A. M. , Clipp, S. L. , Helzlsouer, K. J. , & Platz, E. A. (2010). Association between plasma total cholesterol concentration and incident prostate cancer in the CLUE II cohort. Cancer Cause Control, 21(1), 61–68. 10.1007/s10552-009-9434-8 PMC3004752 19806465

[jev212042-bib-0032] Murphy‐Ullrich, J. E. , & Hook, M. (1989). Thrombospondin modulates focal adhesions in endothelial cells. Journal of Cell Biology, 109(3), 1309–1319. 10.1083/jcb.109.3.1309 PMC2115751 2768342

[jev212042-bib-0033] Murphy‐Ullrich, J. E. , Lightner, V. A. , Aukhil, I. , Yan, Y. Z. , Erickson, H. P. , & Hook, M. (1991). Focal adhesion integrity is downregulated by the alternatively spliced domain of human tenascin. Journal of Cell Biology, 115(4), 1127–1136. 10.1083/jcb.115.4.1127 PMC2289958 1720121

[jev212042-bib-0034] Paila, Y. D. , & Chattopadhyay, A. (2010). Membrane cholesterol in the function and organization of G‐protein coupled receptors. Sub‐Cellular Biochemistry, 51, 439–466. 20213554 10.1007/978-90-481-8622-8_16

[jev212042-bib-0035] Pantel, K. (2010). Bone marrow micrometastasis and circulating tumor cells in cancer patients. Tumor Biology, 31, S2–S2.

[jev212042-bib-0036] Peinado, H. , Alečković, M. , Lavotshkin, S. , Matei, I. , Costa‐Silva, B. , Moreno‐Bueno, G. , … Lyden, D. (2012). Melanoma exosomes educate bone marrow progenitor cells toward a pro‐metastatic phenotype through MET. Nature Medicine, 18(6), 883–891. 10.1038/nm.2753 PMC3645291 22635005

[jev212042-bib-0037] Peinado, H. , Zhang, H. Y. , Matei, I. R. , Costa‐Silva, B. , Hoshino, A. , Rodrigues, G. , … Lyden, D. (2017). Pre‐metastatic niches: organ‐specific homes for metastases. Nature Reviews Cancer, 17(5), 302–317. 28303905 10.1038/nrc.2017.6

[jev212042-bib-0038] Platz, E. A. , Clinton, S. K. , & Giovannucci, E. (2008). Association between plasma cholesterol and prostate cancer in the PSA era. International Journal of Cancer, 123(7), 1693–1698. 18646186 10.1002/ijc.23715 PMC2536746

[jev212042-bib-0039] Platz, E. A. , Till, C. , Goodman, P. J. , Parnes, H. L. , Figg, W. D. , Albanes, D. , … Kristal, A. R. (2009). Men with low serum cholesterol have a lower risk of high‐grade prostate cancer in the placebo arm of the prostate cancer prevention trial. Cancer Epidem Biomar, 18(11), 2807–2813. 10.1158/1055-9965.EPI-09-0472 PMC2877916 19887582

[jev212042-bib-0040] Plebanek, M. P. , Angeloni, N. L. , Vinokour, E. , Li, J. , Henkin, A. , Martinez‐Marin, D. , … Volpert, O. V. (2017). Pre‐metastatic cancer exosomes induce immune surveillance by patrolling monocytes at the metastatic niche. Nature Communications, 8(1), 1319. 10.1038/s41467-017-01433-3 PMC5673063 29105655

[jev212042-bib-0041] Plebanek, M. P. , Bhaumik, D. , Bryce, P. J. , & Thaxton, C. S. (2018). Scavenger receptor type B1 and lipoprotein nanoparticle inhibit myeloid‐derived suppressor cells. Molecular Cancer Therapeutics, 17(3), 686–697. 29282300 10.1158/1535-7163.MCT-17-0981 PMC5935575

[jev212042-bib-0042] Plebanek, M. P. , Mutharasan, R. K. , Volpert, O. , Matov, A. , Gatlin, J. C. , & Thaxton, C. S. (2015). Nanoparticle targeting and cholesterol flux through scavenger receptor type B‐1 inhibits cellular exosome uptake. Scientific Reports, 5, 15724. 26511855 10.1038/srep15724 PMC4625174

[jev212042-bib-0043] Rink, J. S. , Sun, W. , Misener, S. , Wang, J.‐J. , Zhang, Z. J. , Kibbe, M. R. , … Thaxton, C. S. (2018). Nitric oxide‐delivering high‐density lipoprotein‐like nanoparticles as a biomimetic nanotherapy for vascular diseases. Acs Applied Materials & Interfaces, 10(8), 6904–6916. 29385802 10.1021/acsami.7b18525 PMC8495904

[jev212042-bib-0044] Roato, I. , D'Amelio, P. , Gorassini, E. , Grimaldi, A. , Bonello, L. , Fiori, C. , … Ferracini, R. (2008). Osteoclasts are active in bone forming metastases of prostate cancer patients. Plos One, 3(11), e3627. 18978943 10.1371/journal.pone.0003627 PMC2574033

[jev212042-bib-0045] Rosen, C. J. , Ackert‐Bicknell, C. , Rodriguez, J. P. , & Pino, A. M. (2009). Marrow fat and the bone microenvironment: developmental, functional, and pathological implications. Crit Rev Eukar Gene, 19(2), 109–124. 10.1615/critreveukargeneexpr.v19.i2.20 PMC2674609 19392647

[jev212042-bib-0046] Sheng, R. , Chen, Y. , Gee, H. Y. , Stec, E. , Melowic, H. R. , Blatner, N. R. , … Cho, W. (2012). Cholesterol modulates cell signaling and protein networking by specifically interacting with PDZ domain‐containing scaffold proteins. Nature Communications, 3, 1249. 10.1038/ncomms2221 PMC3526836 23212378

[jev212042-bib-0047] Shiozawa, Y. , Eber, M. R. , Berry, J. E. , & Taichman, R. S. (2015). Bone marrow as a metastatic niche for disseminated tumor cells from solid tumors. BoneKEy Reports, 4, 689. 26029360 10.1038/bonekey.2015.57 PMC4440229

[jev212042-bib-0048] Simons, K. , & Ehehalt, R. (2002). Cholesterol, lipid rafts, and disease. Journal of Clinical Investigation, 110(5), 597–603. 10.1172/JCI16390 PMC151114 12208858

[jev212042-bib-0049] Smith, M. R. , Saad, F. , Coleman, R. , Shore, N. , Fizazi, K. , Tombal, B. , … Goessl, C. (2012). Denosumab and bone‐metastasis‐free survival in men with castration‐resistant prostate cancer: results of a phase 3, randomised, placebo‐controlled trial. Lancet (London, England), 379(9810), 39–46. 10.1016/S0140-6736(11)61226-9 PMC3671878 22093187

[jev212042-bib-0050] Sottnik, J. L. , & Keller, E. T. (2013). Understanding and targeting osteoclastic activity in prostate cancer bone metastases. Current Molecular Medicine, 13(4), 626–639. 23061677 10.2174/1566524011313040012 PMC3624036

[jev212042-bib-0051] Steck, T. L. , & Lange, Y. (2018). Transverse distribution of plasma membrane bilayer cholesterol: picking sides. Traffic (Copenhagen, Denmark), 19(10), 750–760. 10.1111/tra.12586 29896788

[jev212042-bib-0052] Svensson, K. J. , Christianson, H. C. , Wittrup, A. , Bourseau‐Guilmain, E. , Lindqvist, E. , Svensson, L. M. , … Belting, M. (2013). Exosome uptake depends on ERK1/2‐heat shock protein 27 signaling and lipid Raft‐mediated endocytosis negatively regulated by caveolin‐1. The Journal of Biological Chemistry, 288(24), 17713–17724. 23653359 10.1074/jbc.M112.445403 PMC3682571

[jev212042-bib-0053] Takegahara, N. , Kim, H. , Mizuno, H. , Sakaue‐Sawano, A. , Miyawaki, A. , Tomura, M. , … Choi, Y. (2016). Involvement of receptor activator of nuclear factor‐kappaB ligand (RANKL)‐induced incomplete cytokinesis in the polyploidization of osteoclasts. The Journal of Biological Chemistry, 291(7), 3439–3454. 26670608 10.1074/jbc.M115.677427 PMC4751386

[jev212042-bib-0054] Thery, C. , Witwer, K. W. , Aikawa, E. , Alcaraz, M. J. , Anderson, J. D. , Andriantsitohaina, R. , & … Zuba‐Surma, E. K. (2018). Minimal information for studies of extracellular vesicles 2018 (MISEV2018): a position statement of the International Society for Extracellular Vesicles and update of the MISEV2014 guidelines. J Extracell Vesicles, 7(1), 1535750. 30637094 10.1080/20013078.2018.1535750 PMC6322352

[jev212042-bib-0055] Thysell, E. , Surowiec, I. , Hornberg, E. , Crnalic, S. , Widmark, A. , Johansson, A. I. , … Wikstrom, P. (2010). Metabolomic characterization of human prostate cancer bone metastases reveals increased levels of cholesterol. Plos One, 5(12), e14175. 21151972 10.1371/journal.pone.0014175 PMC2997052

[jev212042-bib-0056] Van Hemelrijck, M. , Walldius, G. , Jungner, I. , Hammar, N. , Garmo, H. , Binda, E. , … Holmberg, L. (2011). Low levels of apolipoprotein A‐I and HDL are associated with risk of prostate cancer in the Swedish AMORIS study. Cancer Cause Control, 22(7), 1011–1019. 10.1007/s10552-011-9774-z 21562751

[jev212042-bib-0057] Wang, H. F. , Leng, Y. M. , & Gong, Y. P. (2018). Bone marrow fat and hematopoiesis. Front Endocrinol, 9, 694. 10.3389/fendo.2018.00694 PMC6280186 30546345

[jev212042-bib-0058] Weidle, U. H. , Birzele, F. , Kollmorgen, G. , & Ruger, R. (2016). Molecular mechanisms of bone metastasis. Cancer Genomics & Proteomics, 13(1), 1–12. 26708594

[jev212042-bib-0059] Yang, S. , Damiano, M. G. , Zhang, H. , Tripathy, S. , Luthi, A. J. , Rink, J. S. , … Thaxton, C. S. (2013). Biomimetic, synthetic HDL nanostructures for lymphoma. Proceedings of the National Academy of Sciences of the United States of America, 110(7), 2511–2516. 23345442 10.1073/pnas.1213657110 PMC3574906

[jev212042-bib-0060] Yu, S. H. , Liu, C. R. , Su, K. H. , Wang, J. H. , Liu, Y. L. , Zhang, L. M. , … Zhang, H. G. (2007). Tumor exosomes inhibit differentiation of bone marrow dendritic cells. Journal of Immunology, 178(11), 6867–6875. 10.4049/jimmunol.178.11.6867 17513735

[jev212042-bib-0061] Zheng, H. , Li, W. , & Kang, Y. (2016). Tumor‐stroma interactions in bone metastasis: molecular mechanisms and therapeutic implications. Cold Spring Harbor Symposia on Quantitative Biology, 81, 151–161. 28381439 10.1101/sqb.2016.81.030775

