# Supplementary material for: Prostate cancer extracellular vesicles mediate intercellular communication with bone marrow cells and promote metastasis in a cholesterol‐dependent manner
Source: J Extracell Vesicles. 2020 Dec 31;10(2):e12042. doi: 10.1002/jev2.12042 (PMC7775568; doi:10.1002/jev2.12042)
Supplement: Supplementary file 1 — Supporting Information [file JEV2-10-e12042-s001.pdf]

Supplementary Material accompanying:

Prostate cancer extracellular vesicles mediate intercellular communication with bone marrow cells and promote metastasis in a cholesterol-dependent manner

Stephen E. Henrich,[1,2] Kaylin M. McMahon, [1,2] Michael P. Plebanek,[1,2] Andrea E. Calvert,[1,2] Timothy J. Feliciano,[1,2] Sam Parrish,[1] Fabio Tavora,[6] Anthony Mega,[4,5] Andre De Souza,[4,5] Benedito A. Carneiro,[4,5*] and C. Shad Thaxton[1,2,3*]

[1]Department of Urology, Feinberg School of Medicine; [2]Simpson Querrey Institute for BioNanotechnology; and [3]Robert H. Lurie Comprehensive Cancer Center, Northwestern University, 303 E. Chicago Ave., Chicago, Illinois, USA

[4]Warren Alpert Medical School, Brown University, [5]Lifespan Cancer Institute, Providence, Rhode Island, USA

[6]Department of Pathology, Messejana Heart and Lung Hospital, Fortaleza, Brazil.

Supporting Figures: S1-S7

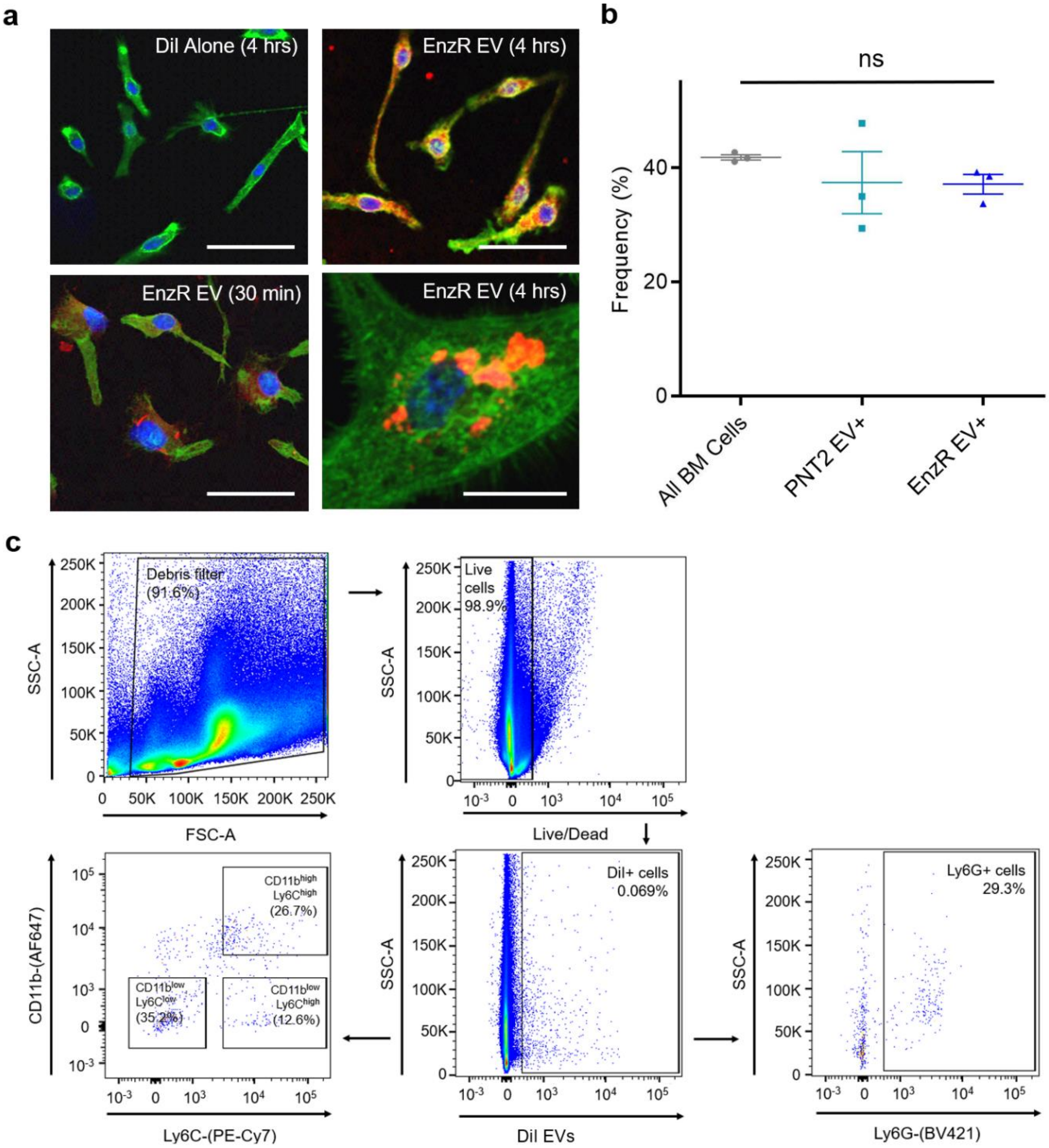

Fig. S1 | EnzR EVs are uptaken by primary cultures of mouse BMMs *in vitro* and do not exhibit preferential targeting of granulocytes in mouse bone marrow *in vivo*. a) Immunocytochemistry of primary mouse BMM cultures treated with DiI-labeled EnzR EVs (red). Far right panel is under high magnification (100X). Green: Phalloidin. Blue: DAPI. Scale bars are 50 µm (top left, top right, and bottom left panels) and 20 µm (bottom right panel). b) Quantification of the frequency of Ly6G$^+$ cells in total bone marrow vs. EnzR and PNT2 EV-targeted cells. c) Sample gating scheme for EV targeting experiments in mouse bone marrow. One-way analysis of variants (ANOVA) with Tukey's method, two-sided was used to determine statistical significance. NS, non-significant. Data are mean ± s.e.m.

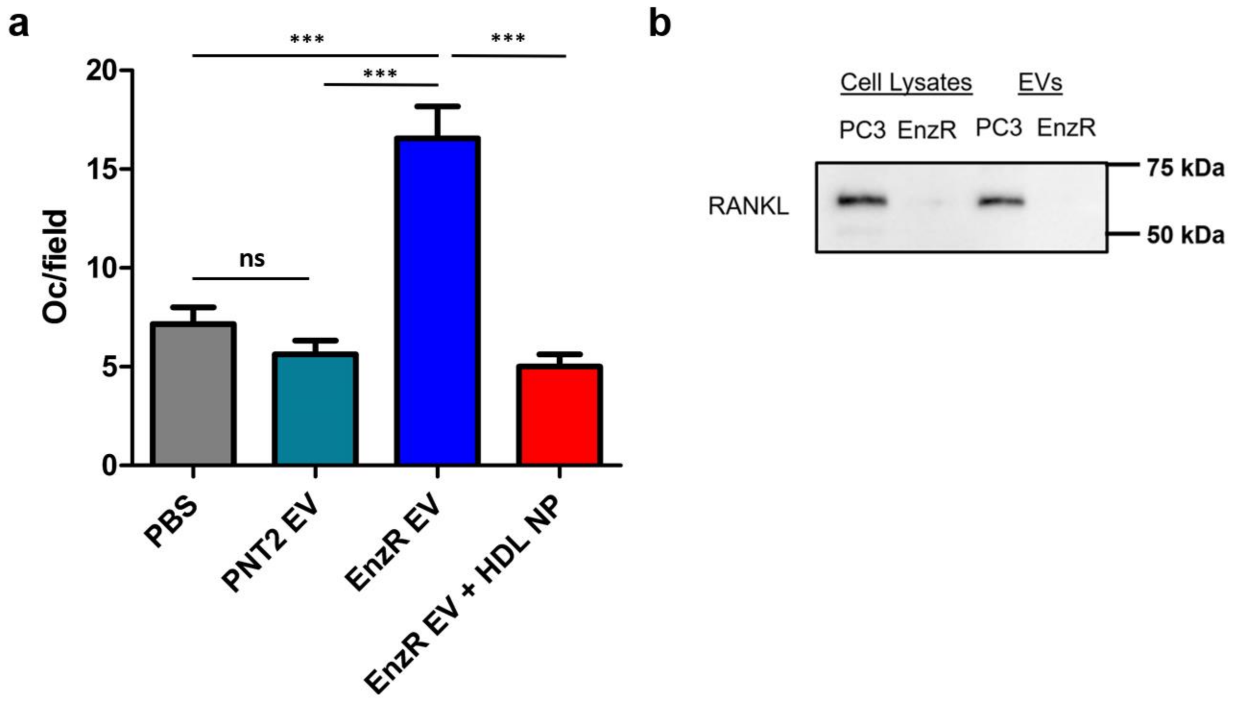

Fig. S2 | EnzR EVs promote osteoclast differentiation *in vivo* in a cholesterol-dependent manner, and do not express RANKL. a) Quantification of *in vivo* osteoclast differentiation assay. Mouse hind limb tissue sections were stained with TRAP to identify and quantify osteoclasts (Oc) (TRAP+, multinucleated cells). b) Immunoblot of EnzR EVs and PC3 EVs (positive control) for RANKL. One-way analysis of variants (ANOVA) with Tukey's method, two-sided was used to determine statistical significance. NS, non-significant. *** $P < 0.001$. Data are mean ± s.e.m.

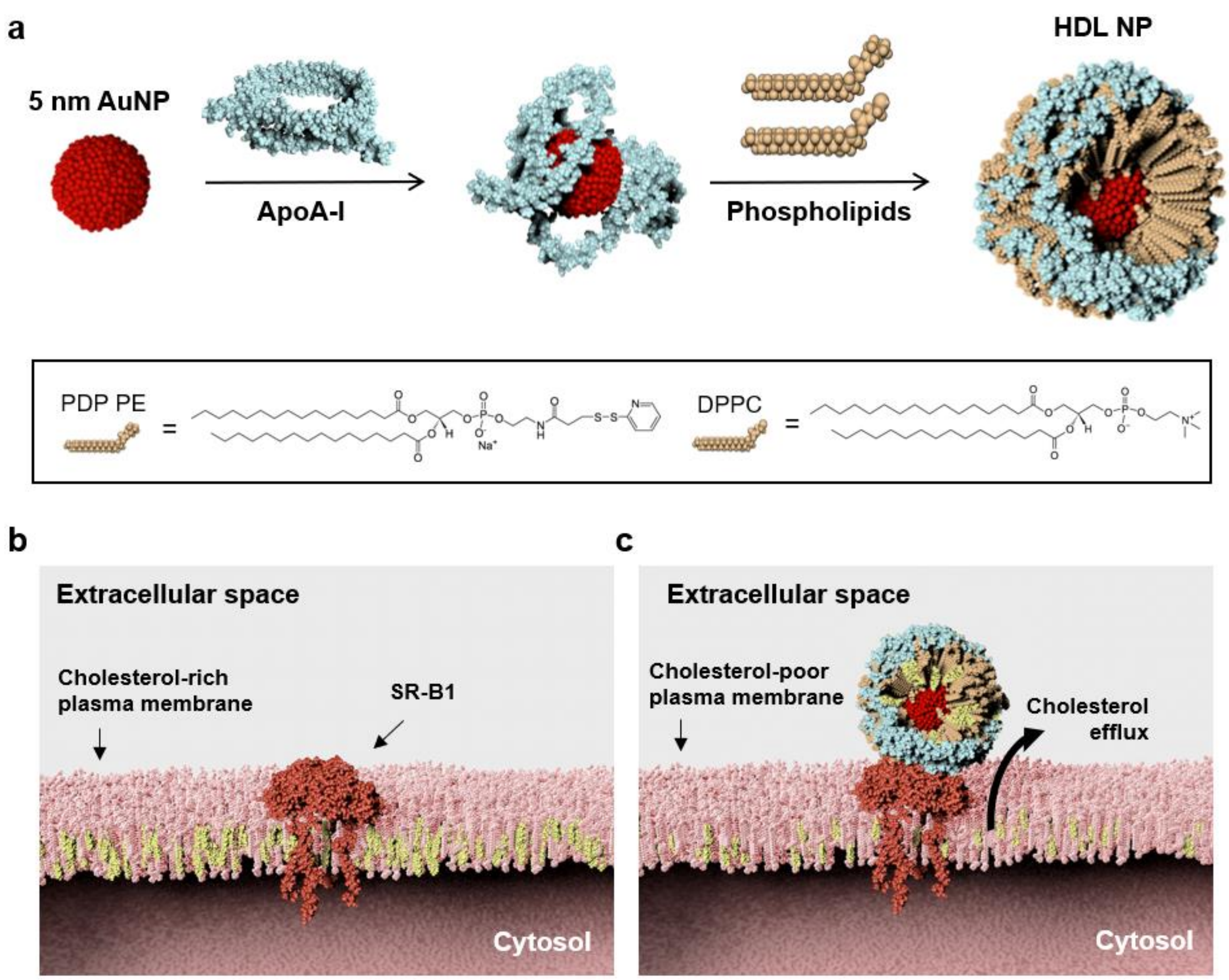

Fig. S3 | HDL NP synthesis scheme and mechanism of cholesterol reduction. a) Synthesis of HDL NPs is accomplished using a 5 nm diameter gold nanoparticle (AuNP) as a core scaffold. Purified apolipoproteinA-1 (apoA-1) is added to the AuNP core scaffold to confer the cell-specific targeting properties and cholesterol efflux capabilities of native HDL. Finally, two species of phospholipids-- 1,2-dipalmitoyl-*sn*-glycero-3-phosphoethanolamine-*N*-[3-(2-pyridyldithio)propionate] (PDP PE) and 1,2-dipalmitoyl-*sn*-glycero-3-phosphocholine (DPPC) -- are added to stabilize the resulting particle structure and to achieve HDL-mimicking surface chemistry. b) Illustration of the cholesterol-rich plasma membrane of target bone marrow myeloid cells, with the endogenous HDL receptor, SR-B1, displayed at the cell surface. c) Illustration of HDL NPs binding to SR-B1 on the surface of bone marrow myeloid cells and reducing cellular cholesterol via cholesterol efflux. Cholesterol transit from the cell membrane onto the surface of HDL particles occurs via hydrophobic channels embedded in the SR-B1 receptor.[61]

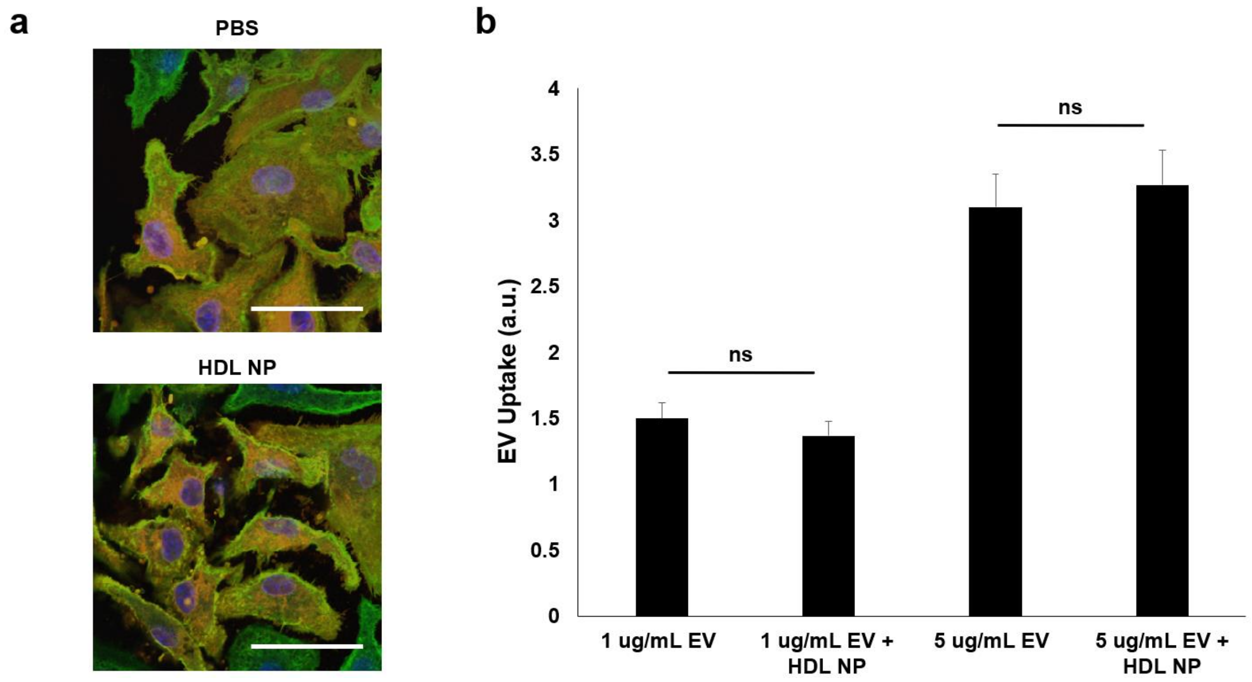

Fig. S4 | HDL NP-mediated inhibition of EnzR EV uptake requires SR-B1. a) Immunocytochemistry of primary mouse BMM cultures harvested from SR-B1$^{-/-}$ mice treated with DiI-labeled EnzR EV (red). Green: Phalloidin. Blue: DAPI. Scale bars are 50 µm. b) Quantification of EnzR EV uptake in BMMs harvested from SR-B1$^{-/-}$ mice, with and without HDL NP treatment. One-way ANOVA with Tukey's method, two-sided was used to determine significance. NS, non-significant. Data are mean ± s.e.m.

.

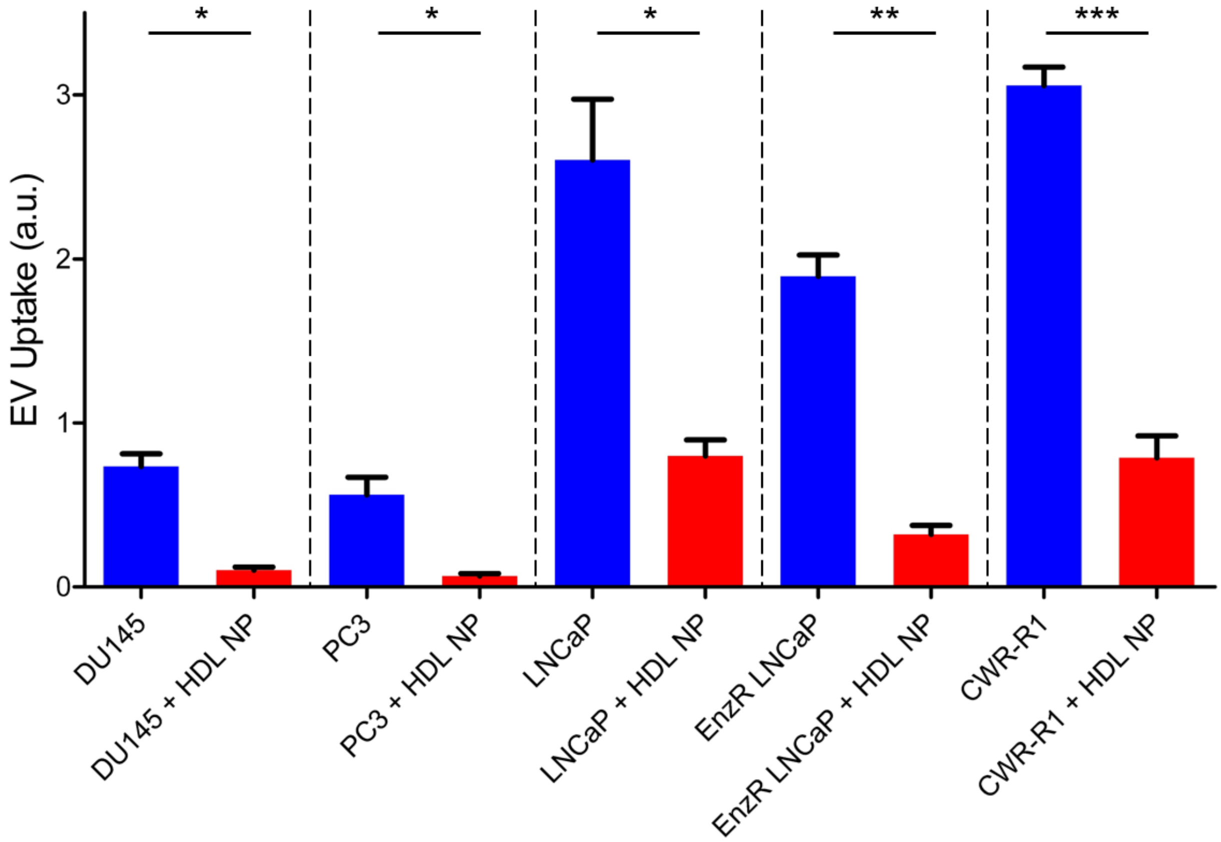

Fig. S5 | HDL NPs inhibit *in vitro* cellular uptake of PCa EVs derived from multiple PCa cell lines. Quantification of EV uptake as determined by confocal fluorescence microscopy of mouse BMM primary cultures treated with DiI-labeled PCa EVs with or without HDL NP pre-treatment (100 nM). Two-tailed Welch's *t*-test was used to determine significance. *$P < 0.05$, **$P < 0.01$, *** $P < 0.001$. Data are mean ± s.e.m.

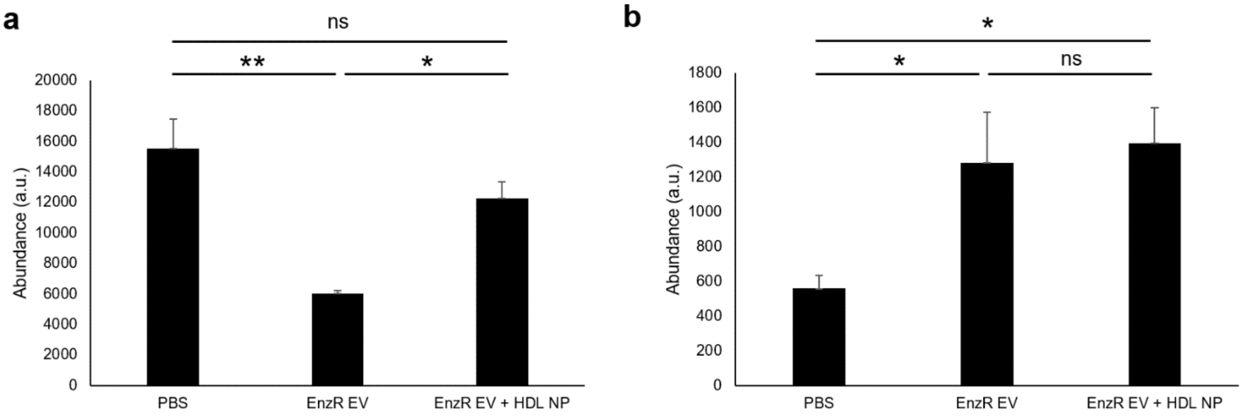

Fig. S6 | Systemic injection of HDL NPs prevents EnzR EV-mediated reduction of bone marrow myeloid cell TSP1 expression but does not prevent enhancement of VCAN expression. RNA expression of a) TSP1 and b) VCAN in $CD11b^+$ bone marrow myeloid cells harvested from mice conditioned with EnzR EVs with or without HDL NP pre-treatment vs. vehicle (PBS) controls. One-way ANOVA with Tukey's method, two-sided was used to determine significance. NS, non-significant; *$P < 0.05$, **$P < 0.01$. Data are mean ± s.e.m.

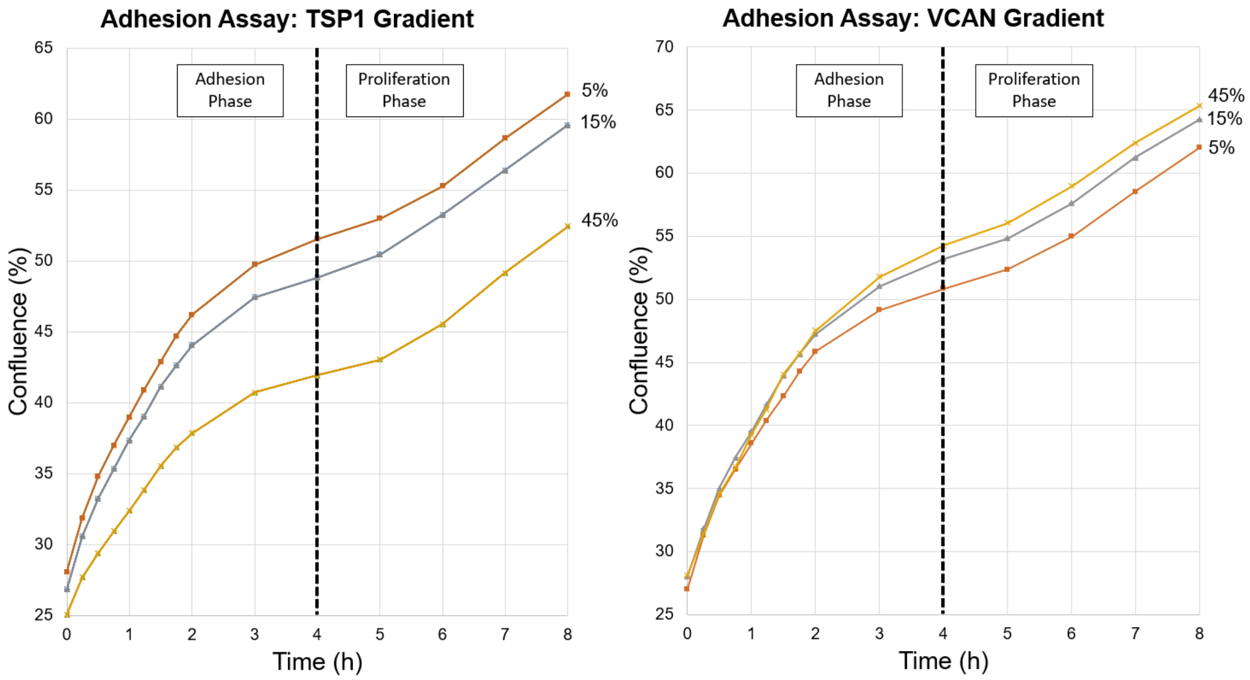

Fig. S7 | Confluence plots for live cell imaging of EnzR CWR-R1 cells introduced into tissue culture wells coated with two-dimensional ECM substrates reveals distinct adhesion and proliferation phases. Confluence plots for EnzR CWR-R1 cells added to collagenous ECM substrates with varying TSP1 (left panel) and VCAN (right panel) content over 8 h, indicating that adhesion primarily occurs within the first 4 h.
